# Postexercise Lactate Clearance, *T*_2_ Relaxation, and *J* ‐Modulation in Human Skeletal Muscle Measured With Double‐Quantum Filtered ^1^H MRS at 7 T

**DOI:** 10.1002/mrm.70295

**Published:** 2026-02-10

**Authors:** Kostiantyn Repnin, Vasco Rafael Rocha dos Santos, Veronika Cap, Roberta Frass‐Kriegl, Siegfried Trattnig, Graham J. Kemp, Martin Meyerspeer

**Affiliations:** ^1^ High Field MR Center, Center for Medical Physics and Biomedical Engineering Medical University of Vienna Vienna Austria; ^2^ High Field MR Center, Department of Biomedical Imaging and Image‐Guided Therapy Medical University of Vienna Vienna Austria; ^3^ Department of Musculoskeletal and Ageing Science and Liverpool Magnetic Resonance Imaging Centre (LiMRIC) Liverpool UK

**Keywords:** dipolar coupling, double‐quantum filter, in vivo, lactate, single‐voxel spectroscopy, skeletal muscle, *T*_2_ relaxation time

## Abstract

**Purpose:**

^1^H MRS lactate measurements are potentially valuable for studying energy metabolism in working skeletal muscle, but some technical obstacles need to be overcome. Spectral filtering to isolate the lactate signal from overlapping lipid resonances shows promise. We report a novel sequence with 3D localisation and a CH‐selective double quantum filter (DQF) which enables dynamic postexercise single‐shot measurement of cellular lactate *T*_2_ and clearance kinetics.

**Methods:**

Using a two‐channel ^1^H transceiver coil at 7 T we applied a localized CH‐selective DQF sequence to postexercise calf muscle in 11 subjects. In one leg acquisitions with constant and incrementing *T*_E_ were interleaved to measure lactate decrease and *T*_2_. In the other the CH‐selective sequence was interleaved with the nonselective variant.

**Results:**

Postexercise DQF shows lactate clearly in 4 s single‐shot spectra. Fitting was able to separate lactate concentration kinetics from *T*_2_ and *J*‐modulation: clearance *t*_1/2_ = 162 ± 42 s and *T*_2_ = 138 ± 20 ms (mean ± SD); the scalar coupling constant fitted from time evolution was *J =* 16.6 ± 0.8 Hz, closely matching *J =* 16.5 ± 1.3 Hz derived from spectral splitting. Direct comparison showed 2.3 ± 0.9 times higher signal with the new CH‐selective sequence.

**Conclusions:**

The new sequence improved lactate detection, enabling quantification from single shots postexercise with time resolution similar to ^31^P MRS. Measuring clearance and relaxation time constants of intramuscular lactate lays the groundwork for future absolute quantification and studies of intra‐ and extracellular lactate compartmentation based on dipolar coupling differences.

## 1 Introduction

Lactate's importance in metabolism [1] goes beyond the old concept of an anaerobic waste product in intense exercise [2]. Lactate circulates between organs as a carbohydrate fuel [3], and inter‐ and intracellular lactate shuttles are important in energy metabolism, redox biology, and cell signaling [4]. In exercising muscle, lactate is produced by reduction of pyruvate generated glycolytically in anaerobic ATP synthesis, whose integration with ATP production by mitochondrial oxidative phosphorylation, “ATP buffering” via creatine kinase, and cellular lactate/H^+^ efflux is still incompletely understood [1, 5]. To study these, dynamic measurement of intracellular metabolites is helpful. ^31^P MRS is useful [6], particularly to study oxidative ATP production [7], but quantitative ^31^P MRS‐based analysis of glycolytic ATP synthesis and cellular H^+^ handling depends on somewhat indirect inference [8]. Direct ^1^H MRS quantitation of lactate with high time resolution, particularly combined with ^31^P MRS, would be a valuable tool to study the physiology of high‐intensity exercising muscle.

Although some work has been done [9, 10, 11, 12], lactate measurement in vivo poses considerable technical challenges. One is sensitivity. Lactate in skeletal muscle is ˜1 mM at rest, increasing ˜30‐fold [2] in intense exercise. Even at high concentrations, the sample volume must be a substantial portion of a moderate‐to‐large muscle in, for example, thigh or calf.

A second challenge is overlapping signal from lipids, largely triglyceride [13]. To suppress lipids, various *J*‐editing schemes have been proposed [9]. A promising approach is double‐quantum filtering (DQF), which can provide higher time resolution than *J*‐difference editing and is less demanding on per‐scan frequency correction to mitigate cancellation errors, because in each single‐shot acquisition only the target resonance passes the filter, albeit at the cost of half the signal. Two DQF variations have been reported for lactate. A 90_x_—180_y_—90_x_—90_CH_—180_y_ scheme containing two refocusing pulses, a hard *x*‐pulse (nonselective, i.e., active both on CH and CH_3_) for entering double‐quantum (DQ) and zero‐quantum (ZQ) states, and a CH‐selective read pulse [10] was the basis of a 3D‐localized PRESS implementation [11, 12, 14]. An alternative sequence, Sel‐MQC [15], in which CH_3_ is selectively excited and a CH‐selective pulse is used both to enter and exit DQ and ZQ states, was implemented as 1D and CSI versions [16, 17].

A third challenge arises from muscle fiber orientation effects, via dipolar coupling [18, 19], on apparent lactate splitting. Optimal echo spacing in a DQF sequence therefore depends on the angle of the fibers to the *B*_0_ field. In previous work [12, 14] fiber orientation in gastrocnemius muscle (24°–35°) was accounted for by calculating an expected effective *J*, *T*_2_ was taken from animal studies [20], and echo time (*T*_E_) was set to compromise values of 57 [12] and 84 ms [14].

A fourth challenge is posed by animal muscle ex vivo evidence [20, 21] for intracellular and extracellular lactate compartments with different *T*_2_: it is unknown whether these exist in vivo and undergo different dipolar coupling and apparent splitting. Dynamic measurements of lactate relaxation in human muscle have been attempted with STEAM [22] quantifying CH directly and CH_3_ by subtracting pre‐ from postexercise spectra with 32 s time resolution; however, the *T*_2_ calculation was hampered by *J*‐phase and STEAM effects [23], leaving only two *T*_E_ points for fitting. For DQF, timings of the first refocusing part of the sequence can be varied to introduce *T*_2_‐weighting without phase modulation due to *J*‐coupling, instead causing amplitude modulation. Selective refocusing eliminates amplitude modulation by keeping *J*‐evolution time fixed during the first refocusing part, and this has been used to measure lactate *T*_2_ [21, 24]. We have not followed this approach, however, because if extracellular and intracellular lactate do have different coupling constants, a pulse sequence sensitive to *J*‐coupling could potentially exploit these to investigate their distribution.

Because lactate concentration falls quickly after high‐intensity exercise, single‐shot acquisitions are desirable to estimate *T*_2_ from spectra acquired with multiple echo times, especially as increasing echo time causes periodic signal modulation depending on the scalar coupling constant, which depends on fiber orientation relative to *B*_0_, due to dipolar effects [19].

Here we present a novel sequence with 3D localization and a CH‐selective DQF part similar to Sel‐MQC, which enables dynamic postexercise measurements of lactate in human calf with unprecedented single‐shot time resolution, allowing dynamic measurement of lactate *T*_2_ and a first insight into the compartmentation problem in vivo.

## 2 Theory

### 2.1 DQF: Density Matrix Formalism

DQF exploits coupling interaction between spins. The classical multiquantum filtering (MQF) experiment can be described using a density matrix basis set expansion [25]. Using product operators [26], we start with magnetization in the *z* direction *I*_*z*_, *S*_*z*_ (the only nonzero product operator terms, for CH and CH_3_ spins). During the preparation part of the sequence *τ*_1_, which precedes the MQF part, *J*‐coupling product operator transformations take place, leaving nonzero antiphase terms of *I*_*z*_ *S*_*x*_, *I*_*x*_ *S*_*z*_ in the basis set.

### 2.2 Effects of Different Coupling Systems and *x‐*Pulses

This assumes that the IS_3_ system (i.e., one proton (I) coupled with three magnetically equivalent protons (S_3_)—a weakly coupled four‐spin system) can be treated as an IS system (one proton (I) coupled with one other proton (S)—a weakly coupled two‐spin system), which is not strictly true. For an IS_3_ system a hard *x‐*pulse transforms the spin system to a superposition of DQ and ZQ coherences, because of asymmetric evolution of CH and CH_3_ spin terms during the *J*‐coupling cycle. At exactly 0.5/*J* preparation time the product operator terms for an IS_3_ system, arising from the initial CH_3_ and CH terms, are *I*_*z*_ *S*_*x*_ *S*_0_ *S*_0_, *I*_*z*_ *S*_0_ *S*_*x*_ *S*_0_, *I*_*z*_ *S*_0_ *S*_0_ *S*_*x*_, and *I*_*x*_ *S*_*z*_ *S*_*z*_ *S*_*z*_, of which the first three (only) can be simplified to *I*_*z*_ *S*_*x*_. When the preparation time is not exactly 0.5/*J*, there are also intermediate terms *I*_*x*_ *S*_*z*_ *S*_0_ *S*_0_, *I*_*x*_ *S*_0_ *S*_*z*_ *S*_0_, *I*_*x*_ *S*_0_ *S*_0_ *S*_*z*_, *I*_*y*_ *S*_*z*_ *S*_*z*_ *S*_0_, *I*_*y*_ *S*_*z*_ *S*_0_ *S*_*z*_, *I*_*y*_ *S*_0_ *S*_*z*_ *S*_*z*_, three of which contribute to DQ + ZQ states when a hard *x*‐pulse is played out. This complicates outcome prediction [21], especially where the preparation period—*τ*_1_ is varied, as in our experiments to measure *T*_2_.

### 2.3 Hard Versus CH‐Selective Pulses

A CH‐selective pulse acting only on the *I* spin will affect only terms of the type *I*_*z*_ *S*_*x*_ (*I*_*z*_ *S*_*x*_ *S*_0_ *S*_0_, *I*_*z*_ *S*_0_ *S*_*x*_ *S*_0_, *I*_*z*_ *S*_0_ *S*_0_ *S*_*x*_) converting them to ZQ + DQ coherence terms *I*_*y*_ *S*_*x*_ (*I*_*x*_ *S*_*y*_ *S*_0_ *S*_0_, *I*_*x*_ *S*_0_ *S*_*y*_ *S*_0_, *I*_*x*_ *S*_0_ *S*_0_ *S*_*y*_) or *I*_*x*_ *S*_*x*_, (*I*_*x*_ *S*_*x*_ *S*_0_ *S*_0_, *I*_*x*_ *S*_0_ *S*_*x*_ *S*_0_, *I*_*x*_ *S*_0_ *S*_0_ *S*_*x*_), depending on the phase of the pulse.

Notably, the phase of the CH‐selective entry pulse has no practical influence on DQF. This is in contrast to a hard *x* entry pulse, where any phase deviation impairs DQF efficiency, leading, for example, to voxel‐position‐dependent effects related to the spins' phase relative to the phase of the hard entry pulse [11, 27].

## 3 Methods

### 3.1 Exercise and Measurement Protocol

**FIGURE 1:**
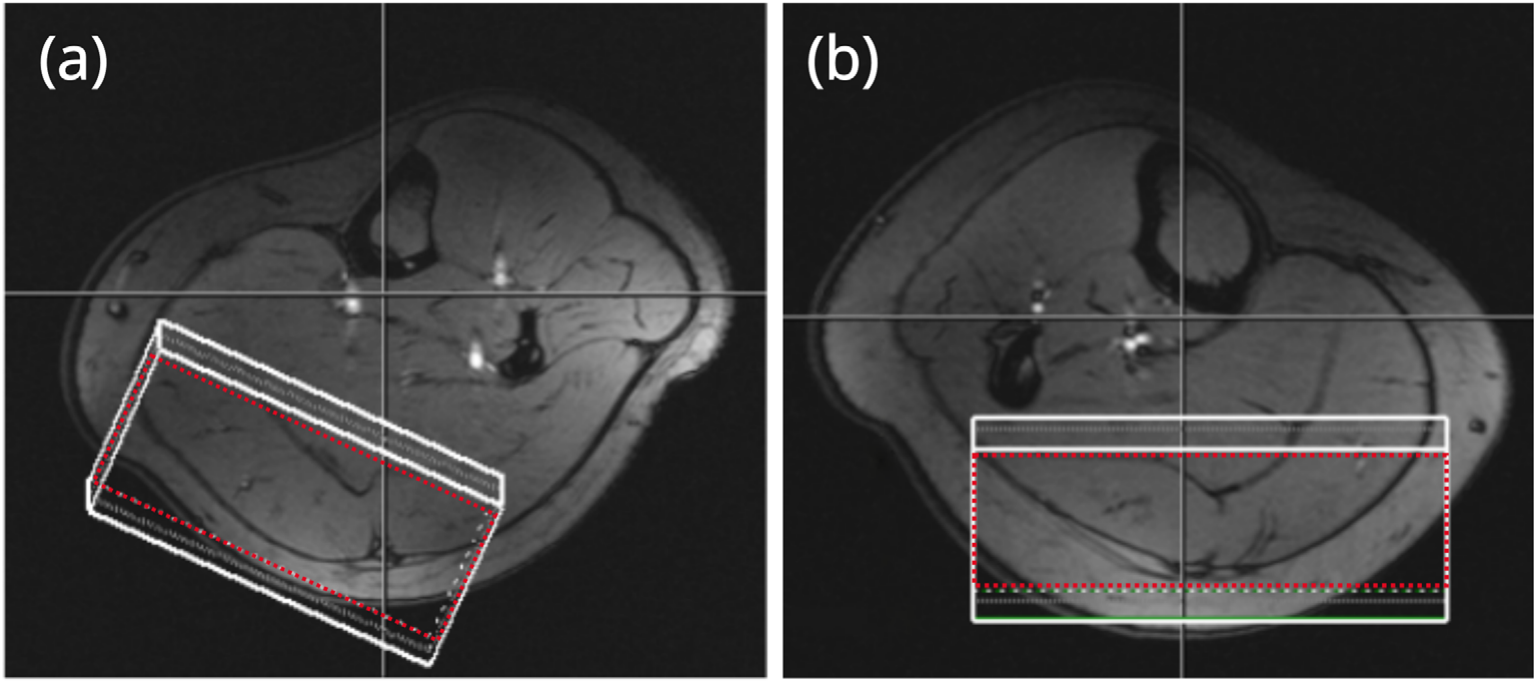
Typical MRS voxels in (a) left and (b) right leg, shown on transverse GRE localizer images. Average voxel volume was 205 cm^3^ (typically c. 8 × 3 × 8 cm in HF‐AP‐RL directions). The effective voxel (red) accounts for the chemical shift displacement artifact, reducing the volume by 17%.

In vivo measurements used a 7 T whole‐body MRI scanner (Magnetom 7 T Plus, Siemens, Erlangen, Germany) and the proton channels of an in‐house three channel ^31^P, two channel ^1^H transceiver calf coil [28]. Eleven subjects were studied (median age 24 years [range 18–45 years]; six females, five males; median BMI 20 kg/m^2^ [range 16–24 kg/m^2^]) measuring in both legs in consecutive experiments. Subjects provided written fully‐informed consent in accordance with the Declaration of Helsinki and local ethics approval. Subjects were placed on the table with the calf on the RF coil. Leg position was marked with a cross drawn on nonirritating tape at the position of the light visor, to ensure reproducible positioning before and after exercise. After localizer scans and *B*_1_ adjustment (Figures 1 and S1) DQF spectra were acquired at rest as a baseline to verify suppression of lipids and water. Then subjects left the scanner to perform standing single‐leg calf‐raise (ankle extension) exercise in the scanner room repeated every ˜2 s until exhaustion. Subjects were rapidly re‐positioned in the scanner, using the marker, and *B*_0_ shimming performed, allowing MRS acquisition ˜1 min after the end of exercise. The protocol was repeated using the other leg.

The left leg was used to measure lactate *T*_2_ and postexercise clearance by interleaving the new CH‐selective sequence with constant and incremental sequence timing every 4 s. The right leg was used to compare the new 3D‐localized DQF sequence with the CH nonselective variant [14], interleaving them every 4 s. One subject was re‐invited for additional measurement of *T*_2_ and lactate clearance in both legs. In total 24 datasets were acquired: 13 datasets for *T*_2_ and lactate clearance (from 11 subjects, 12 datasets in left leg, one in right leg), 11 datasets for sequence comparison (right leg only). Each dataset contains two temporally interleaved time‐courses, either for measuring *T*_2_ and lactate clearance or for sequence comparison, with resting‐state spectra acquired before exercise.

### 3.2 The MRS Setup

Localizer scans (10 axial, five sagittal, three coronal slices) were acquired to select the MRS voxel encompassing gastrocnemius muscle (see Figure 1). In the left leg, predominantly gastrocnemius medialis was selected with an oblique voxel (207 ± 77 cm^3^), while in the right leg both gastrocnemius medialis and lateralis were selected, using a near‐coronal voxel orientation (202 ± 82 cm^3^). The voxel size was set to encompass as much of gastrocnemius as possible (typically c. 8 × 3 × 8 cm in HF‐AP‐RL directions), often overlapping with the adjacent head of gastrocnemius and small parts of soleus (see Discussion). Shimming used the manufacturer's standard routine based on a gradient‐echo field mapping sequence, and an axial *B*_1_ field map was acquired using the double‐flip‐angle method [29] to adjust the reference voltage for the MRS voxel.

**FIGURE 2:**
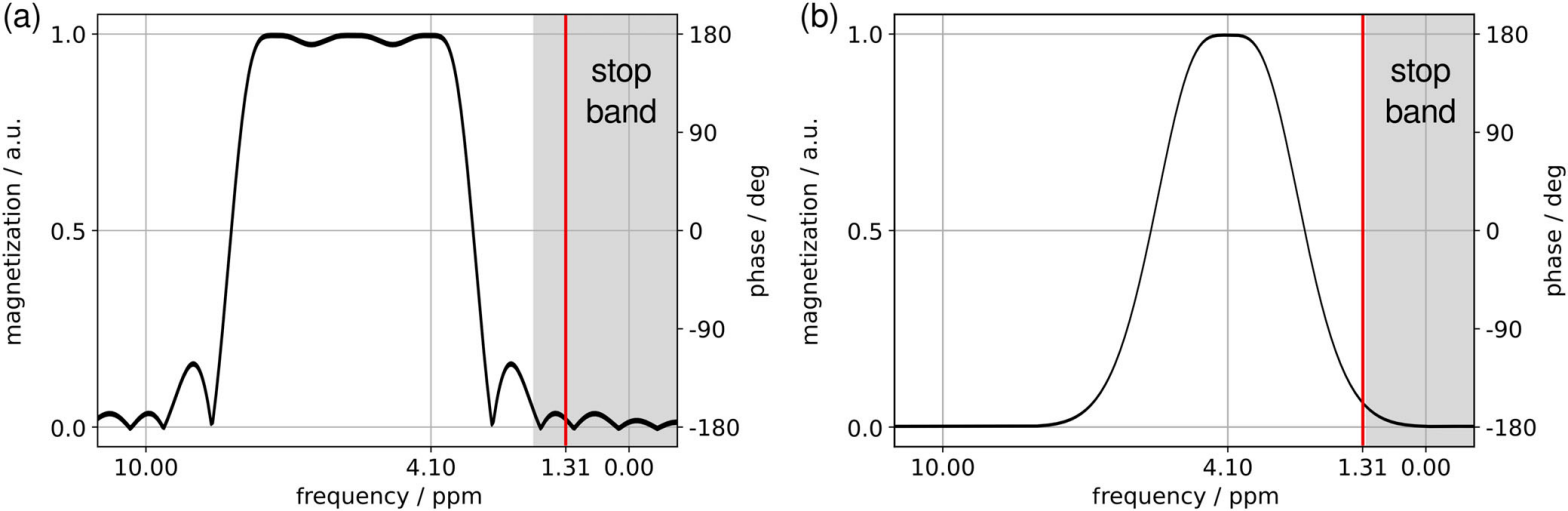
Simulated pulse profiles of (a) a 5‐lobe sinc and (b) a Gaussian pulse for comparison, demonstrating effects on magnetization and bandwidth. The simulation was performed using the density matrix formalism. Absolute magnetization (black) denotes flipped magnetization in simulation of the excitation pulse. Stop‐band regions (shaded) represent < 5% flipped magnetization, according to expert consensus [30]. The Gaussian pulse was centered at 4.1 ppm, the 5‐lobe sinc pulse shifted by 1.63 ppm from 4.1 ppm.

The new sequence was localized in three directions, with slice‐selective excitation (Hamming‐filtered 7‐lobe sinc pulse, duration 2.6 ms, bandwidth (BW) 3.3 kHz) and spatially‐selective adiabatic refocusing pulse‐pairs (smoothed chirp, duration 4.6 ms, BW 5.0 kHz), similar to semi‐LASER with addition of DQF pulses (5‐lobe sinc, duration 4.0 ms, BW 1.7 kHz) acting frequency‐selectively on CH (see Figure 2a). CH selectivity was achieved by centering the carrier frequency at +1.03 ppm from transmitter frequency (i.e., water) so the CH resonance was within the pulse pass‐band [30] (see Figure 2a). The semi‐LASER RF pulses were centered −2.0 ppm from the transmitter frequency.

**FIGURE 3:**
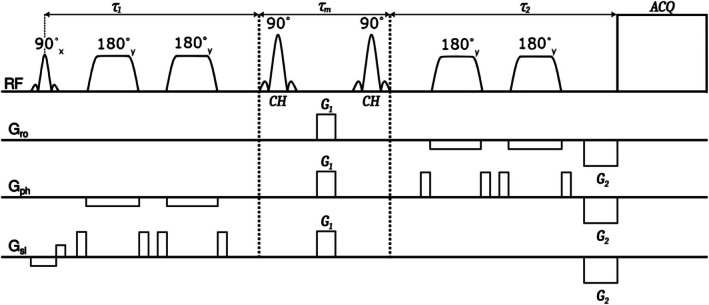
The new CH‐selective MRS sequence: 3D‐localized semi‐LASER with DQF. Total echo time *T*_E_ contains *τ*_1_ and *τ*_*2*_ spin‐echo parts of the semi‐LASER base with unrefocused quantum encoding *τ*_m_ part. Pulses: Excitation (Hamming‐filtered 7‐lobe sinc pulse, duration 2.6 ms, BW 3.3 kHz); adiabatic refocusing (smoothed chirp, duration 4.6 ms, BW 5.0 kHz); DQF entry and exit (5‐lobe sinc, duration 4.0 ms, BW 1.7 kHz). Unbalanced quantum‐filtering gradients *G*_1_ and *G*_2_ have gradient moment ratio of 1:2. Gradient spoilers used together with refocusing pulses had duration of 2.3 ms and gradient amplitude of 15 mT/m. DQF gradient spoilers had duration of 2.3 ms and gradient amplitude of 19/32/32 mT/m in slice/phase/readout directions respectively.

Timings in the *τ*_1_‐modulated sequence were: *τ*_1_ = 22.8 ms (starting value), *τ*_m_ = 10.6 ms and *τ*_2_ = 35 ms. To measure *T*_2_ decay and lactate clearance, the sequence was played out with alternating timings: an acquisition with constant echo delays (to measure pure lactate clearance) was followed by an acquisition with *τ*_1_ incremented by 10 ms, each half‐increment being symmetrically added to each side of the second refocusing pulse (see Figure 3); maximum *τ*_1_ was limited to 500 ms, the sequence continuing to interleave with fixed timings for 20 min in all (300 measurements with *T*_R_ = 4 s). Resting state data were acquired in 50 DQF measurements.

Sequence timings in the method comparison differed from the lactate clearance measurement due to timing limitations in the nonselective DQF sequence: *τ*_1_ = 35 ms, *τ*_m_ = 10.6 ms and 9 ms (in CH‐selective and hard entry sequence respectively) and *τ*_2_ = 40 ms for both sequences compared. Four spectra were acquired at rest, and 32 measurements postexercise.

In a lactate‐containing test object, signal dependence on the phase of the hard DQF entry pulse was compared with the CH‐selective DQF entry pulse. The carrier frequency of the CH‐selective pulse was adjusted, depending on pulse duration, to keep 4.1 ppm on the edge of the pass‐BW (see Figure 2a); this was calculated as (1/duration) × 0.97 × 4, and the carrier frequency was shifted by half of this. Pulse voltage was automatically readjusted when pulse duration changed, keeping nominal flip angle constant.

### 3.3 Spectral Fitting

Spectra were fitted using in‐house python scripts after phasing, zero‐filling and apodization in jMRUI [31, 32]. The lactate doublet was fitted as Voigt‐shaped lines: phases of two peaks, Gaussian and Lorentzian peak widths, ratio of two peaks and splitting constant were held constant through all spectra of a time series; amplitude of the doublet and frequency were adjusted for each timepoint by creating an amplitude‐frequency meshgrid with constant step‐width. The optimal amplitude‐frequency pair was determined using a cost function (sum of the squared point‐wise differences between Voigt functions and data). Optimal Voigt line shape parameters were determined by another iteration. Multiple sets of shape parameters were sampled through uniform randomization and passed in the loop one by one to generate combined cost for the whole time‐series (sum of the each cost per spectrum), using amplitude‐frequency adjustment step, to find the parameter set corresponding to minimal combined cost. Voigt line parameters were determined for each subject individually.

The spectra were frequency‐corrected and zero‐order phase‐adjusted based on the creatine (at 3.03 and 3.91 ppm) and other peaks (at 5.6, 4.5, 4.3, and 2.3 ppm) which pass the DQF. If lipid residues at 1.5 ppm affected the 1.3 ppm region, they were fitted as a Lorentzian and subtracted from the spectrum before fitting lactate as Voigt lines.

### 3.4 Fitting Lactate Kinetic Data

The estimated lactate amplitudes were used to fit the time course. Since two datasets were interleaved, two fitting functions were used: one describes mono‐exponential physiological lactate clearance (Equation 1).

(1) $$A_{\mathrm{rec}}\left(t_{\left[s\right]}\right)=a\cdot \exp\left(\frac{-t_{\left[s\right]}}{t_{\text{clear}}}\right)+b$$

the other the combined effects of lactate clearance, *T*_2_ decay and *J*‐modulation (Equation 2)

(2) $$A_{\text{comb}}\left(t_{\left[\mathrm{ms}\right]}\right)=a\cdot \sin\left(\pi Jt_{\left[\mathrm{ms}\right]}\right)\cdot \exp\left(\frac{-t_{\left[\mathrm{ms}\right]}-\tau_{1}^{\text{fixed}}}{t_{\text{comb}}}\right)+b$$

In these *A*_comb_ and *A*_rec_ are amplitudes of combined and pure clearance decays respectively; *t*_comb_ is combined decay time constant (ms); *t*_clear_ is clearance time constant (s), *t*_[ms]_ is incremented *τ*_1_ timing (ms), *t*_[s]_ is measurement time with a 2*T*_R_ step between data points; $\tau_{1}^{\text{fixed}}$ is 22.8 ms; *a* is initial signal amplitude; *b* is an offset; and *J* is a fitting parameter of amplitude modulation frequency (which should correspond to the apparent splitting of the doublet). The initial signal amplitude was confined within the boundaries of mean ± SD of the five initial data points (corrected for the decay and/or *J*‐modulation using initially guessed values), all other fit parameters being unrestricted.

Assuming both *T*_2_ decay and lactate clearance are mono‐exponential, the *T*_2_ relaxation time constant can be derived from their decay constants (Equation 3).

(3) $$\frac{1}{T_{2}}=\frac{1}{t_{\text{comb}}}-\frac{1}{t_{\text{clear}}}\cdot c_{\left[s\to \mathrm{ms}\right]}$$

These must be brought to a common scale: the combined constant *t*_comb_ is fitted with 10 ms increments and the clearance constant *t*_clear_ with 8 s step width, so the ratio of these sampling intervals is the scaling factor *c*_[s → ms]_ (Equation 4).

(4) $$c_{\left[s\to \mathrm{ms}\right]}=\frac{2\;T_{R}}{\tau_{\mathrm{inc}}}=0.8$$

Fitting was performed in Python using the *curve_fit* method from the *scipy* package.

### 3.5 Method Comparison

To compare the new CH‐selective sequence with the “old” sequence [14], the lactate clearance fit (Equation 1) was carried out on both datasets. In the first iteration all three parameters were unrestricted, then the mean lactate clearance time was set as a fixed parameter of the second iteration, equal for both datasets. The ratio between the resulting amplitudes (parameter *a* in Equation 1) was calculated.

### 3.6 Signal‐to‐Noise Ratio (SNR) Calculations

The single‐shot SNR was calculated for each of the first 10 postexercise spectra (10 Hz apodisation, 2 × zero filling) as the ratio of the lactate peak amplitude to the standard deviation in the artifact‐free region at 7–9 ppm, then averaged across the spectra.

### 3.7 Lipid Suppression

The lipid suppression factor was calculated as the ratio of peak areas at 1.51 ± 0.1 ppm acquired with semi‐LASER (*τ*_1_ = 22.8 ms, *τ*_2_ = 35 ms, 1 average) and the corresponding DQF sequence (*τ*_1_ = 22.8 ms, *τ*_m_ = 10.6 ms, *τ*_2_ = 35 ms, 50 averages) acquired at rest in four subjects (two males, two females, subject Nos. 2, 4, 9, 10, left leg).

### 3.8 Statistical Analysis and Exclusion Criterion

Small sample size (≤ 13) precluded normality tests. We provide mean ± SD (standard deviation) for quantitative findings. We used Mann–Whitney U test to compare *J* values derived from time‐course and spectral splitting. For the kinetic fittings (≤ 150) we used bootstrapping with 50% downsampling and 1000 iterations to provide a 95% confidence interval and estimate fitting variability. The error of the fitting parameter taken as the 95% percentile from 1000 subsamples was used as a criterion to exclude lactate clearance *t*_1/2_ and *T*_2_ with errors exceeding 3·SD of bootstrapping error (four datasets).

## 4 Results

### 4.1 Lactate Postexercise Time‐Course

**FIGURE 4:**
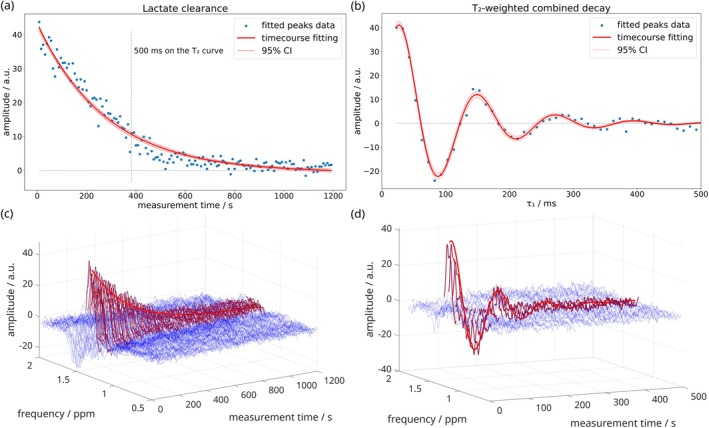
Postexercise lactate kinetics in a single subject. Upper panels are time‐courses of lactate amplitudes showing (a) pure lactate clearance and (b) the combined effect of postexercise clearance, *J‐*modulation and *T*_2_ relaxation, acquired from Subject No. 5 in an interleaved manner with the new sequence. Blue data points represent the amplitude of peaks fitted as Voigt lines, red shows the fit of the amplitude time courses within 95% confidence intervals. Lower panels (c) and (d) show the corresponding spectra as stack plots with 2× zero‐filling and 10 Hz apodisation. Single‐shot acquisitions are shown without averaging with time resolution 8 s (2 × *T*_*R*_ = 2 × 4 s). The red region at 1.3 ± 0.1 ppm highlights the lactate peak, overlaid with the corresponding fits from (a) and (b).

Lactate was detected in DQF spectra postexercise in at least one leg of all 11 subjects. Time‐series of spectra and fitted lactate intensities from a single subject are shown in Figure 4 (time‐courses for *T*_2_ and *J* quantification are in the Figures S2–S8). Physiological clearance of lactate over 20 min is evident in the constant‐*τ*_1_ spectra acquired every 8 s (Figure 4a). The interleaved spectra, incrementing *τ*_1_ from 22.8 to 500 ms, show also the effects of *T*_2_ decay and *J*‐modulation (Figure 4b).

**FIGURE 5:**
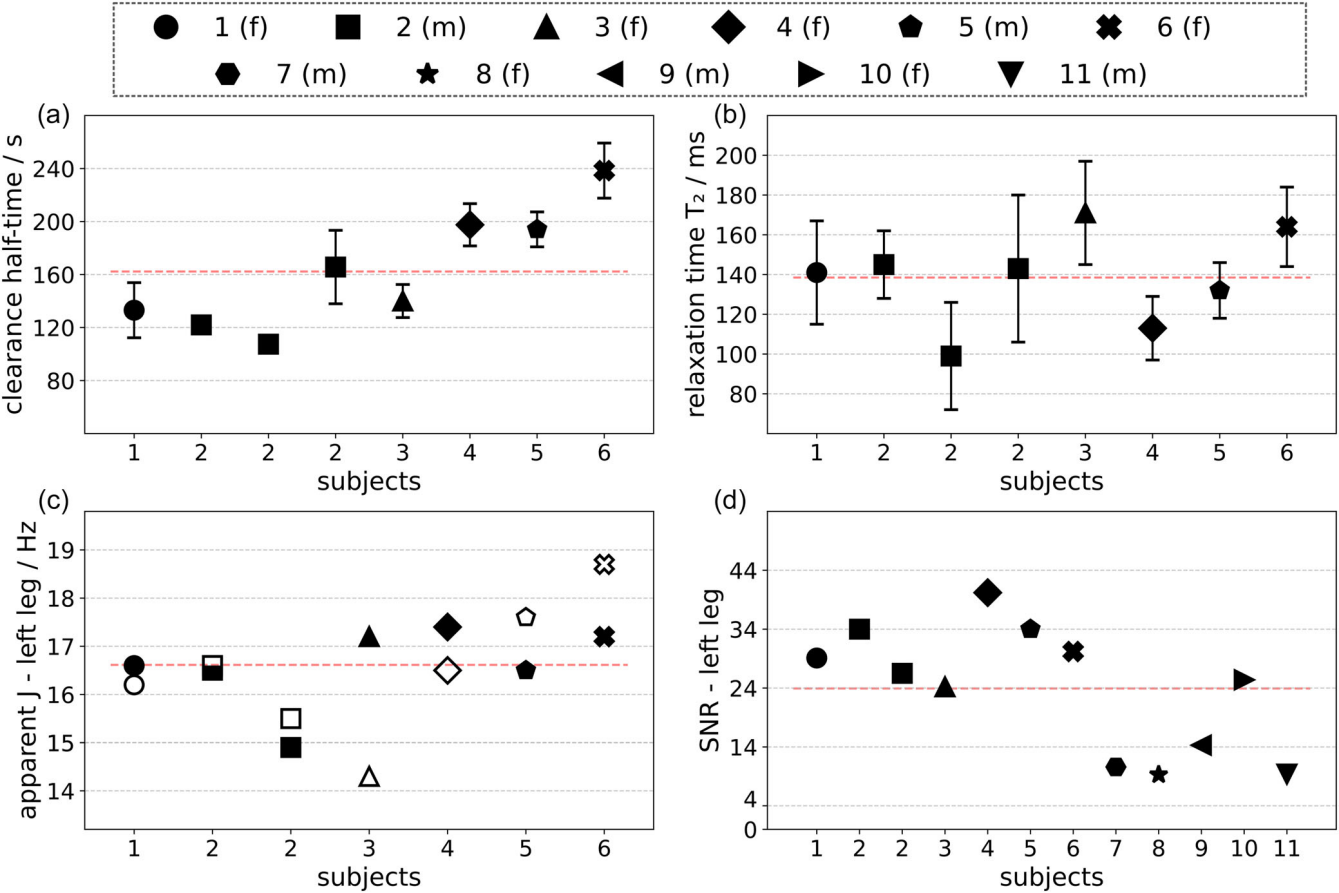
Derived kinetic parameters across subjects using the new sequence. Upper panels show lactate clearance *t*_1/2_ (a) and apparent *T*_2_ (b) in individual subjects. Red dashed lines denote the average. Error bars represent 95% confidence interval, obtained via bootstrapping. Apparent *J* values from left leg are shown in (c), derived from splitting of the doublet (open markers) and the periodicity of the fitted time‐courses (closed markers). SNR of lactate is shown in (d). Plot legend symbols: f, female; m, male.

Lactate clearance half‐times (*t*_1/2_) and apparent *T*_2_ were measured in eight datasets from six subjects (Figure 5a,b). Clearance *t*_1/2_ (mean ± SD) was 162 ± 42 s (median: 153 s) and *T*_2_ 138 ± 20 ms (median: 142 ms). Of 13 datasets acquired to quantify *T*_2_ and clearance time, four did not meet the criterion for fit reliability (error < 3 SD, that is, 32 s for *t*_1/2_ and 46 ms for *T*_2_), and one was excluded due to motion. Subject numbering was reordered accordingly.

SNR in all 11 subjects in the left leg was 23.9 ± 10.6 (median: 26.0).

### 4.2 Apparent Lactate *J*‐Coupling

Apparent lactate *J* was determined in two ways: from amplitude modulation under variation of *τ*_1_ via Equation (2), and from peak splitting in the DQF spectra. In the left leg, amplitude‐modulation gave *J* = 16.6 ± 0.8 Hz (mean ± SD), not significantly different (*U* = 27.5, *p* = 0.748) from the peak‐splitting estimate of *J* = 16.5 ± 1.3 Hz (mean ± SD). In the right leg the best estimate from peak‐splitting (averaging new and old sequences where applicable, before averaging over subjects) was *J* = 17.9 ± 2.5 Hz (mean ± SD). When doublets were unresolved (presumably due to *B*_0_^+^ inhomogeneities), data were omitted from analysis and figures.

### 4.3 Other Metabolite Peaks

In the DQF ^1^H spectra creatine at 3.03 ppm and its coupling partner at 3.91 ppm were visible, as well as peaks at 4.5 and 4.3 ppm (presumably dipolar coupled water) and other peaks at 2.3 ppm and at 5.6 ppm (see Figure S9).

### 4.4 Lipid Suppression

A median lipid suppression factor of 5877 (min = 1300, max = 8760, *n* = 4) was achieved with DQF gradient moments of 226 ms × mT/m, which provided sufficient fitting reliability.

### 4.5 Lactate Method Comparison

**FIGURE 6:**
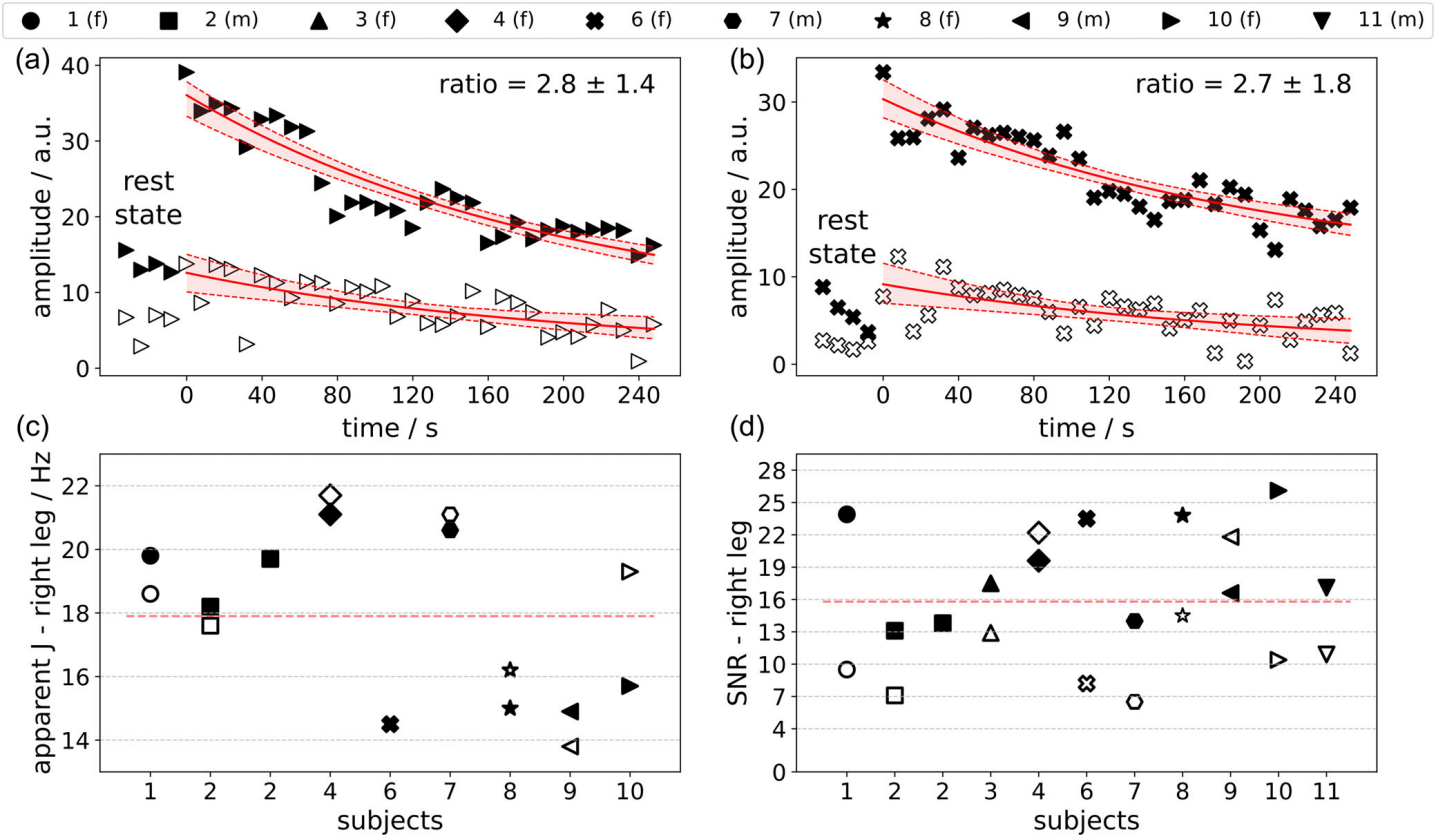
Comparison of new and old sequences in interleaved acquisitions. Upper panels show fitted lactate time‐course in right leg from (a) subject No. 10 and (b) subject No. 6. In all panels filled markers denote the new CH‐selective sequence, open markers the nonselective sequence [14]. For signal‐yield comparison the ratio of fitted amplitudes is given in each top right corner. Filled space between dashed fit lines represents 95% confidence interval, obtained via bootstrapping. The lower panels show individual derived parameters across subjects. Apparent *J* values from splitting of the doublet are shown in (c) (omitted where the doublet was not clearly observed). The SNR of lactate is shown in (d). Red dashed lines denote averages. Plot legend symbols: f, female; m, male.

Using the two sequences (in the right leg), lactate was detected in 10 subjects (see Figure 6). The ratio between amplitudes of the time‐course fits of two sequences (see Methods, Section 3.5) was 2.3 ± 0.9 (mean ± SD) in favor of the new sequence.

In a lactate phantom (160 mM) the new sequence yielded 2.7× higher SNR. In vivo lactate SNR was 1.5× higher with the new sequence: 19.0 ± 4.7 (mean ± SD, median: 17.5) compared to 12.4 ± 5.6 (median: 10.7). The between‐sequence difference in signal varied between subjects. SNR varies more with the nonselective (CV = 45%) than with the CH‐selective sequence (CV = 24%). Of 11 sequence‐comparison datasets one was not used due to technical error in acquisition.

### 4.6 Test Object Studies: Hard Versus CH‐Frequency‐Selective DQF Entry Pulse

**FIGURE 7:**
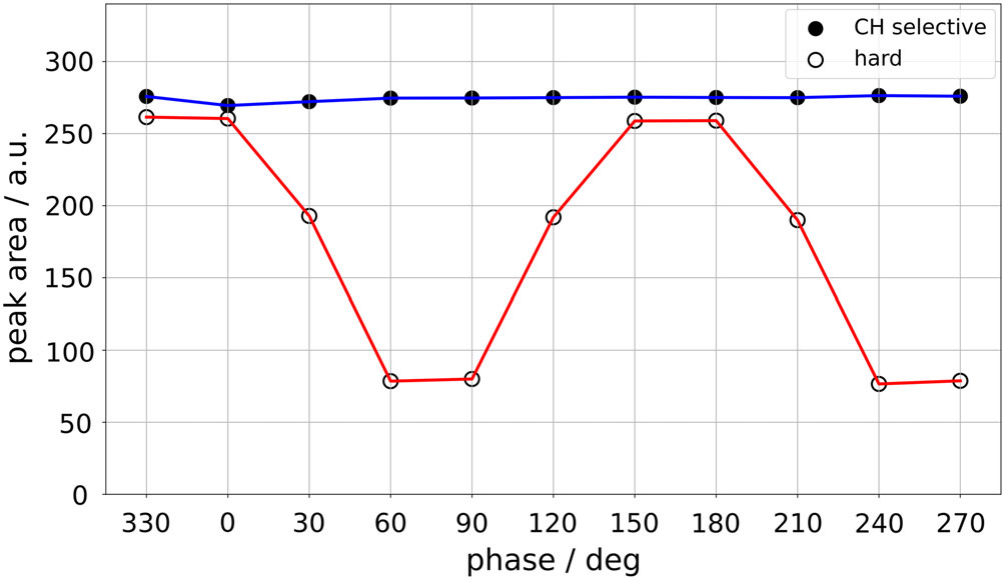
Peak areas of CH_3_ lactate signal acquired in a 160 mM lactate phantom with variation of DQF entry pulse phase. The DQF entry pulse was either CH‐selective (blue) or hard, that is, affecting both CH and CH_3_ (red).

The test object experiments show the independence of the signal from pulse phase (Figure 7) for the selective entry pulse, but sine‐like modulations for the hard pulse.

### 4.7 Lactate Line‐Shape

**FIGURE 8:**
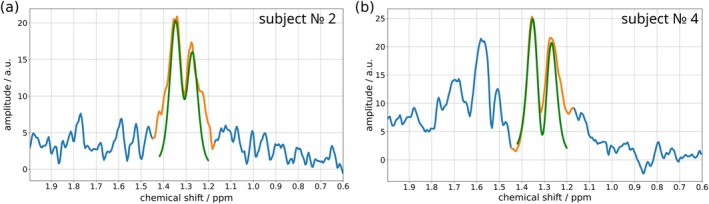
Example of peak fitting and apparent *J* splitting in vivo due to dipolar coupling. The real part of spectra acquired in the right leg of subjects No. 2 (a) and No. 4 (b): *J* splitting 18.2 and 21.1 Hz, respectively. Orange denotes the region used for peak fitting, and green the result of fitting a constant‐shape Voigt function, with amplitude and frequency adjusted to minimize the cost function. The spectra shown are the first ones in the series acquired after exercise. SNR is 15.2 and 21.4, respectively.

A clearly resolved lactate doublet, consistently more pronounced in the right leg due to higher dipolar splitting and longer *τ*_2_ timing (+5 ms compared to left leg, due to limited *B*_1_^+^), which affects the relative phase of the peaks, is shown in Figure 8, together with a demonstration of the constant Voigt‐shape line‐fitting method.

## 5 Discussion

### 5.1 Single‐Shot Lactate Kinetics

We achieved average lactate SNR of 23.9 ± 10.6 after intense calf‐raise exercise outside the magnet, followed by rapid repositioning and shimming. Interleaving measurements with variable and constant *T*_E_ allows fitting *T*_2_ and (orientation‐dependent) apparent *J‐*coupling constant, in addition to physiological lactate clearance rate. Achieving sufficient SNR in single shots is challenging given the DQF techniques required to suppress lipids resonating at the same frequency as lactate CH_3_. Lactate signal intensity varied considerably between subjects, as expected in such strenuous exercise, where lactate accumulation depends on subject compliance. This however cannot affect quantification of *T*_2_, *J* and clearance or sequence comparisons, as data were acquired interleaved after a single exercise bout. Overall, the new CH‐selective sequence showed better lipid suppression and a lower coefficient of variation than the nonselective variant.

Previously‐reported dynamic lactate measurements in vivo in human muscle [14, 22, 33, 34] were acquired with a best temporal resolution of 30 s. Here we achieved a time resolution of 4 s. Ren et al. [22] took a simpler approach using STEAM, but did not account for the expected strong modulation of *J*‐coupled systems, depending on *T*_E_ and *T*_M_ [23]. Ren et al. [22] measured CH which gives less signal than CH_3_ due to its fewer protons and quadruplet splitting. CH_3_ subtraction methods degrade temporal resolution (requiring two spectra per time point) and are prone to subtraction errors caused by the strong lipid signal, mitigated using subscan frequency and phase correction. Prominent motion artifacts are observed during (intermittent) exercise [14, 22], where DQF will be more robust because temporally‐varying partial volume effects or linewidth changes will not cause subtraction artifacts.

### 5.2 Improvements on DQF Entry Pulse

Robust detection by an appropriate DQF sequence is essential. As explained in Section 2.3, conventional DQF is vulnerable to signal loss related to the phase of the hard entry pulse. A CH‐selective DQF entry pulse should avoid this, which is confirmed by our in vivo and test object results. For selective conversion of CH resonances, we followed the approach of shifting a relatively broad‐band pulse with a relatively narrow transition band (580 Hz), allowing a short pulse while preserving high CH selectivity (CH_3−_to‐CH flip angle ratio of 63, cf. 25 for Gaussian pulse, see Figure 2). In general, shorter pulses usefully shorten the DQF part and consequent *J*‐coupling evolution.

### 5.3 Other Metabolites in the Spectrum

The CH‐selective DQF sequence coedits other *J*‐coupled and dipolar coupled metabolites, for example, Cr‐CH_2_ at 3.9 ppm and Cr‐CH_3_ at 3.03 ppm. Very close to lactate CH_3_, the threonine CH_3_ resonates at 1.32 ppm, with a coupling partner at 4.25 ppm [35]. Threonine is an amino acid whose muscle production and efflux rises during intense exercise as a result of proteolysis [36]. However, muscle threonine concentration is only ˜0.1 mM at rest [35, 37], and increases very little in absolute terms during exercise [36], so threonine signal will have negligible effect on lactate clearance and *T*_2_ quantification.

### 5.4 Lipid Suppression

The flip angle of the CH‐selective pulse with duration 4 ms at the CH_3_ frequency of 1.31 ppm was 1.4°. While this is acceptable for the coherence pathway of lactate, it can cause issues for lipid suppression: given the strong lipid signals at 1.33 and 1.5 ppm: even 1% of lipids undergoing the DQ pathway would significantly impact lactate detection. According to a detailed study [38], two main contributors to the 1.33 ppm signal are CH_2_–CH_2_–CH_3_ (I_2_S_3_, I = 1.33 ppm, S = 0.90 ppm; *J* = 8.0 Hz) and CH–CH_2_–CH_2_ (I_2_S_2_, I = 1.33 ppm, S = 2.07 ppm; *J* = 7.14 Hz). It is not clear whether CH selectivity affects lipid suppression solely at 1.33 ppm or whether its coupling partners at 2.07 and 0.90 ppm are also involved. Nevertheless, prolonging these DQF pulses from 3 to 4 ms increased CH selectivity, which improved lipid suppression considerably.

Usefully, for lipids that pass DQF, their coupling constant is ˜2–3 times smaller than the apparent *J* of lactate in vivo [12, 14]. This can help reduce residual lipids by choosing appropriate *τ*_1_. However, in experiments in which *τ*_1_ is modulated, lipid signal might be partially mistaken for extracellular lactate, which is reported to have a peak splitting similar to lactate in solution (*J* = 6.93 Hz) [20]. In our case, lipid suppression improved with increased gradient moments (226 ms × mT/m) and contributed mostly as lipid residue at 1.5 ppm, with phase opposite to the lactate doublet (for *τ*_*1*_ = 22.8 ms and *τ*_2_ = 35 ms). This residual lipid signal was accounted for by fitting and subtraction before lactate quantification. Any residual signal at 1.3 ppm manifested as a constant *y*‐offset (parameter *b* from Equation 1) in the time‐course fitting (see Figures S2–S8). Thus *T*_2_ and clearance rate were little affected by any lipid residue.

### 5.5 Lactate Clearance and *T*_2_ Measurements

Lactate clearance *t*_1/2_ = 2.7 ± 0.7 min (*n* = 8) is in excellent agreement with measurements by muscle biopsy [39] (*t*_1/2_ ˜ 3 min) and the MRS work of Ren et al. [22] (*t*_1/2_ = 2.0 ± 0.6 min, *n* = 12). Pan et al. [34] reported considerably longer clearance times by MRS (*t*_1/2_ = 10.6 min, *n* = 7).

The relaxation time we measured by fitting decay, *J*‐modulation and *T*_2_ in a single interleaved measurement, *T*_2_ = 138 ± 20 ms (*n* = 8), is in good agreement with *T*_2_ = 109 ± 10 ms (*n* = 3) reported by Ren et al. [22] who used a two‐point estimation with *T*_E_ = 100 and 140 ms. A somewhat lower value of *T*_2_ = 80 ms (*n* = 1) was reported by Pan et al. [34] using 4 *T*_E_ points.

The *T*_2_ measurements have some limitations: Since orientation‐dependent dipolar coupling modulates lactate's apparent coupling constant, if the fiber orientation in the voxel is not homogeneous, apparent *J* will be an average of different *J* values; reported *T*_2_ might then underestimate true *T*_2_ due to dephasing of components with different effective *J*. However, our *T*_E_‐modulated data show no evidence of multiple *J* values; also, from measurements in the right leg (where the lactate doublet was resolved better), we conclude that variation of dipolar coupling was small.

Considering the interaction of physiological lactate clearance, *T*_2_ decay and *J*‐modulation, the choice of *τ*_1_ increment is crucial: this controls the extent of *T*_2_‐weighting, which has a major influence on the estimation of *T*_2_. *T*_2_ fitting is unreliable if the signal is insufficiently *T*_2_‐weighted (i.e., *τ*_1_ increment too small) that the combined decay curve is dominated by lactate clearance. Additionally, smaller *τ*_1_ values cover fewer cycles of *J*‐modulation. Based on pilot experiments in an additional *n* = 4 subjects, we chose a time increment of 10 ms (at *T*_R_ = 4 s) as a compromise between dominant *T*_2_‐weighting in the *τ*_1_‐incremented data (larger *τ*_1_ increment leading to fewer data points for *T*_2_ and *J* fitting) and exploiting the points with highest SNR in the early stages of physiological lactate clearance.

DQF spectra acquired in muscle after intense exercise can suffer from motion artifacts, leading to distortion of line shape, lipid contamination or low SNR. Fitting the lactate resonances with AMARES proved challenging (data not shown), as the algorithm frequently did not converge for all spectra in the series using identical starting values and prior knowledge, which precludes consistent quantification throughout the time series. Constant Voigt line shape, derived from the whole time‐course of spectra in a reproducible, cost‐based fitting algorithm, is more robust against individual spectrum line shape variations, which do not represent changes in metabolite concentrations. “True” line shape is imposed by the fitting algorithm, leaving the task to find only the most plausible spectral position and amplitude. Of course, peak widths might vary, but the small fit error (quantified via bootstrapping) of < 10% for the decay rate and < 17% for *T*_2_ and *J* suggest that the fit represents the data well.

Our approach to measuring lactate *T*_2_ dynamically in vivo in the muscle is novel: the only published *T*_2_ data relied on clearance rates measured in separate experiments (which may vary, as discussed above) and used relatively few *T*_E_ points (2 [22] or 7 [34], respectively) for the fit, in contrast to our 48 points.

### 5.6 Lactate Method Comparison

Our in vivo data, supported by test object studies, show that CH‐selective DQF is feasible on a clinical 7 T scanner and yields improved sensitivity with acceptable lipid suppression.

The ratios of spectral SNRs do not fully represent differences in lactate signal yield, due to small lipid residuals that could only be taken into account as offset when fitting the time‐courses. Lipid suppression is better with CH‐selective DQF, as with nonselective DQF, more lipid residues erroneously contribute to the SNR measurement. Overall, the new CH‐selective sequence proved more robust (evidenced in a lower coefficient of variation) than the nonselective variant.

It is likely that inaccurately tuned phase of the nonselective hard DQF entry pulse, pragmatically implemented in previous studies [12, 14, 20, 21, 40], was a factor impairing DQF efficiency in the earlier sequence, and contributed to the inference of low lactate MRS visibility [41]. Besides fitting uncertainty, other sources of signal variability in both sequences include potentially inhomogeneous lactate distribution, different excitation and refocusing frequencies (1.3 vs. 2.8 ppm in the old versus new sequence) and resulting chemical shift displacement artifacts [42]. Notably, the subtraction volume effect in a DQF sequence attributes only to refocusing after the quantum encoding part (see Figure 3), since other coherences do not pass the filter.

Lactate measurements on lower‐field and clinical scanners, to reach comparable temporal resolution, will likely require additional SNR enhancements, for example, using more efficient RF coils [43]. The sequence is based on a standard single‐voxel localization scheme (PRESS), extended with the DQF part and adiabatic refocusing (Figure 3) in Siemens IDEA (version VE12U). It can be implemented by research groups who have access to the sequence programming environment.

### 5.7 Voxel Selection

Our voxel extends beyond the anatomical boundaries of the target muscle, encompassing heterogeneous fiber orientation. We wished to cover a large part of gastrocnemius (which bears the main load in plantar flexion with straight knee [44, 45]), to achieve maximum signal, accepting the inclusion of some soleus and subcutaneous fat. Contributions from soleus are small (if present at all, considering chemical shift displacement, see below), and soleus is expected to exercise less (hence accumulate less lactate, if any) than gastrocnemius in this toe‐raise exercise. Including fat challenges lipid suppression but will not increase true lactate signal.

The effective voxel is 17% smaller than the nominal voxel in the direction of the first pair of refocusing pulses (see Figure 3) due to chemical shift displacement artifact (both lactate resonances need to be inverted at a given position). Slightly different regions were selected in the left and right legs, due to B_1_^+^ asymmetry (Figure S1). Different fiber orientations could lead to contributions from lactate edited with differing efficacy due to differing apparent *J* couplings; this might affect the accuracy of *T*_2_ estimation, but we did not find evidence for substantial *J* variation within the selected volume.

### 5.8 Lactate Compartmentation

*J*‐modulated time‐courses obtained with excellent time resolution and SNR suggest that only one compartment of lactate is detected, and because of the observed splitting this is presumably the intracellular component. A previous study [22] using STEAM also reported one major source of lactate signal, with similar splitting *J* = 19.6 ± 2.3 Hz (in this study 17.2 ± 2.1 Hz, averaged in both legs). Nevertheless it cannot be ruled out that extracellular lactate was present [39] but invisible, due to, for example, diffusion weighting.

The orientation of muscle fibers included in the voxel that partially covered the other head of gastrocnemius and very small fractions of soleus was approximated as a model with single *J*. Despite heterogeneous volume selection, kinetic data acquired in subjects with a sufficiently high SNR and well‐resolved splitting of the doublet does not support strong compartmentation effects arising from partial volume effects.

## 6 Conclusion

This is, we believe, the first report of dynamic measurement of *T*_2_ relaxation time of dipolar‐coupled lactate, in vivo in skeletal muscle. Single‐shot acquisitions will enable future studies to quantify lactate with unprecedented time scale resolution. The *J*‐modulated experiments confirm (within the margin of error) the presence of one major source of the lactate signal, the dipolar‐coupled intracellular compartment. The new sequence avoids the phase incoherence problem that can lead to signal loss in a conventional 3D localized DQF sequence.

## Funding

This work was supported by the Austrian Science Fund (10.55776/P35305).

## Supporting information


**FIGURE S1.** Typical *B*_1_^+^ map acquired from a subject using the double‐flip‐angle method in the left leg.
**FIGURE S2.** Data described in the paragraph above for subject No. 1. The offset, of 9% of the total signal is presumably due to lipid residues at 1.3 ppm.
**FIGURE S3.** Data described in the paragraph above for subject No. 2.
**FIGURE S4.** Data described in the paragraph above for subject No. 2, re‐invited (left leg).
**FIGURE S5.** Data described in the paragraph above for subject No. 2, re‐invited (right leg).
**FIGURE S6.** Data described in the paragraph above for subject No. 3. The offset, of 10% of the total signal is presumably due to lipid residues at 1.3 ppm.
**FIGURE S7.** Data described in the paragraph above for subject No. 4. The offset, of 20% of the total signal is presumably due to lipid residues at 1.3 ppm.
**FIGURE S8.** Data described in the paragraph above for subject No. 6.
**FIGURE S9.** Typical DQF spectrum acquired in the subject No. 4, averaged through the whole time‐course (150 time points) in the lactate clearance dataset only. Spectra were not frequency or phase corrected before averaging, contributing in additional broadening of the peaks. Zero filling was (2×) and apodisation (10 Hz) were applied.

**Data S1.** mrm70295‐sup‐0002‐supinfo.

## Data Availability

All simulation code and parameters are provided at https://github.com/wastehling/T1T2‐mapping‐BBFS.git.
