# Supplementary material for: Postexercise Lactate Clearance, T 2 Relaxation, and J ‐Modulation in Human Skeletal Muscle Measured With Double‐Quantum Filtered 1H MRS at 7 T
Source: Magn Reson Med. 2026 Feb 10;95(6):3065–76. doi: 10.1002/mrm.70295 (PMC13049247; doi:10.1002/mrm.70295)
Supplement: Supplementary file 1 — FIGURE S1. Typical B 1 + map acquired from a subject using the double‐flip‐angle method in the left leg. FIGURE S2. Data described in the paragraph above for subject No. 1. The offset, of 9% of the total signal is presumably due to lipid residues at 1.3 ppm. FIGURE S3. Data described in the paragraph above for subject No. 2. FIGURE S4. Data described in the paragraph above for subject No. 2, re‐invited (left leg). FIGURE S5. Data described in the paragraph above for subject No. 2, re‐invited (right leg). FIGURE S6. Data described in the paragraph above for subject No. 3. The offset, of 10% of the total signal is presumably due to lipid residues at 1.3 ppm. FIGURE S7. Data described in the paragraph above for subject No. 4. The offset, of 20% of the total signal is presumably due to lipid residues at 1.3 ppm. FIGURE S8. Data described in the paragraph above for subject No. 6. FIGURE S9. Typical DQF spectrum acquired in the subject No. 4, averaged through the whole time‐course (150 time points) in the lactate clearance dataset only. Spectra were not frequency or phase corrected before averaging, contributing in additional broadening of the peaks. Zero filling was (2×) and apodisation (10 Hz) were applied. [file MRM-95-3065-s001.pdf]

Supplementary Material

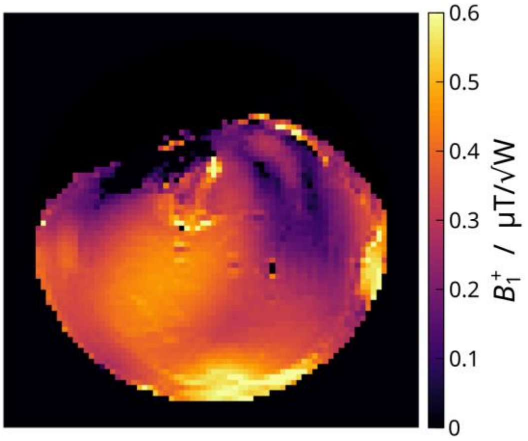

FIGURE S1. Typical B_1^+ map acquired from a subject using the double-flip-angle method in the left leg.

Figures S2-S8: Time-courses of lactate amplitudes from single subjects showing (a) pure lactate clearance and (b) the combined effect of post-exercise clearance, J modulation and T_2 relaxation, acquired in an interleaved manner using the new sequence. Blue data points represent the amplitude of peaks fitted as Voigt lines, red shows the fit of the amplitude time courses and its 95 % confidence intervals. Individual data points (marked with a red cross) were excluded from the fit when they deviated by more than 3 standard deviations from the fit in a first run.

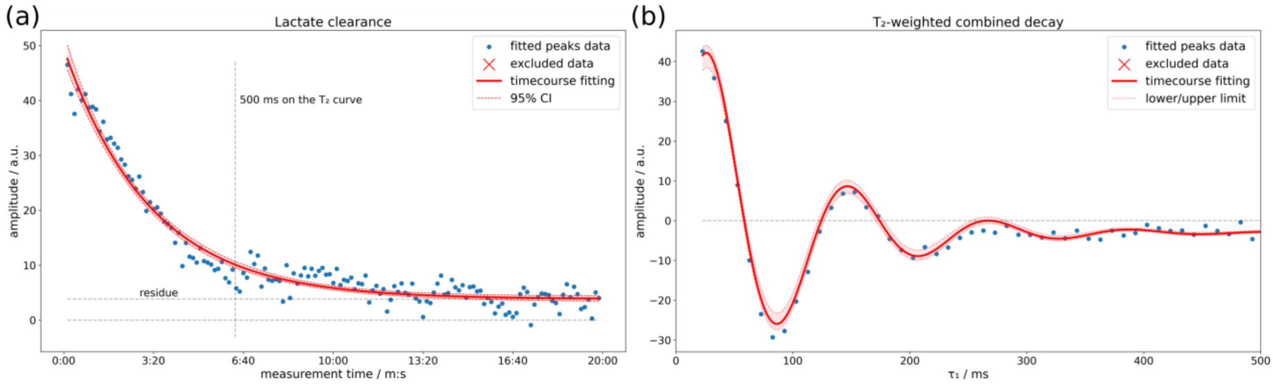

FIGURE S2. Data described in the paragraph above for subject № 1. The offset, of 9 % of the total signal is presumably due to lipid residues at 1.3 ppm.

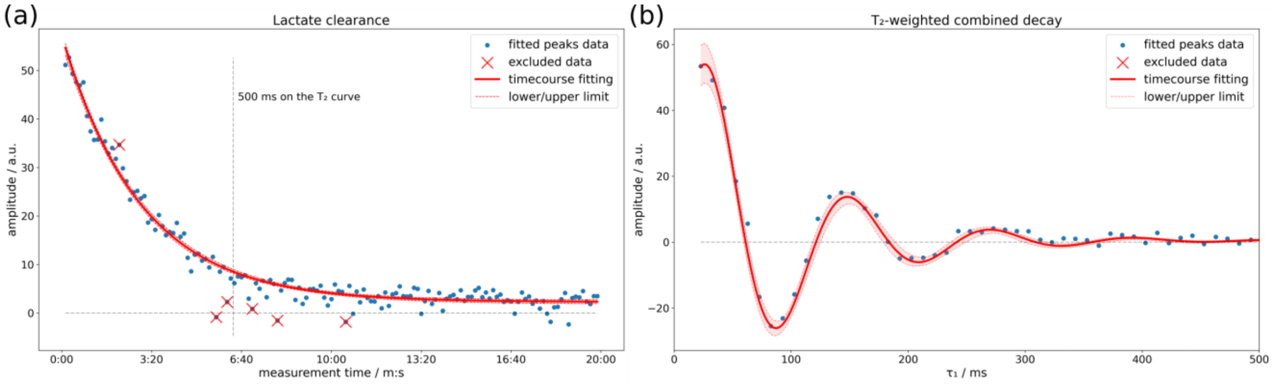

FIGURE S3. Data described in the paragraph above for subject № 2.

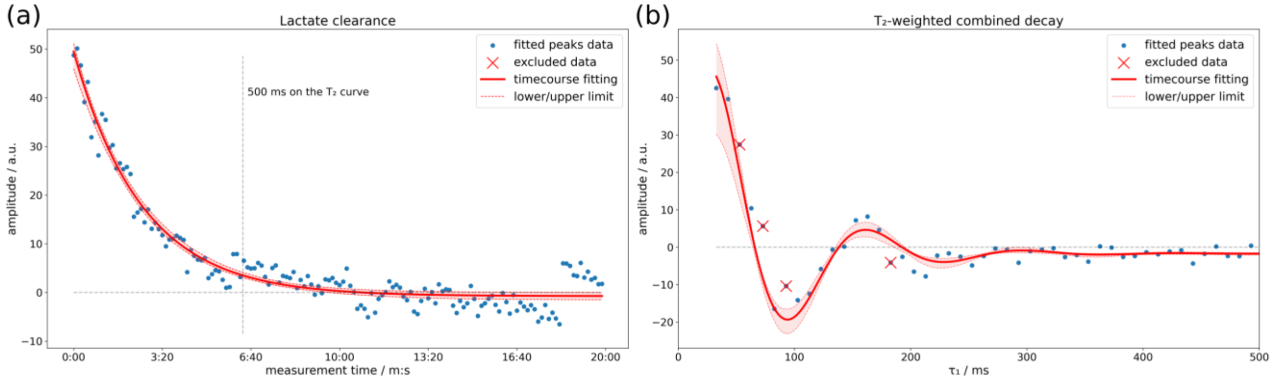

FIGURE S4. Data described in the paragraph above for subject № 2, re-invited (left leg).

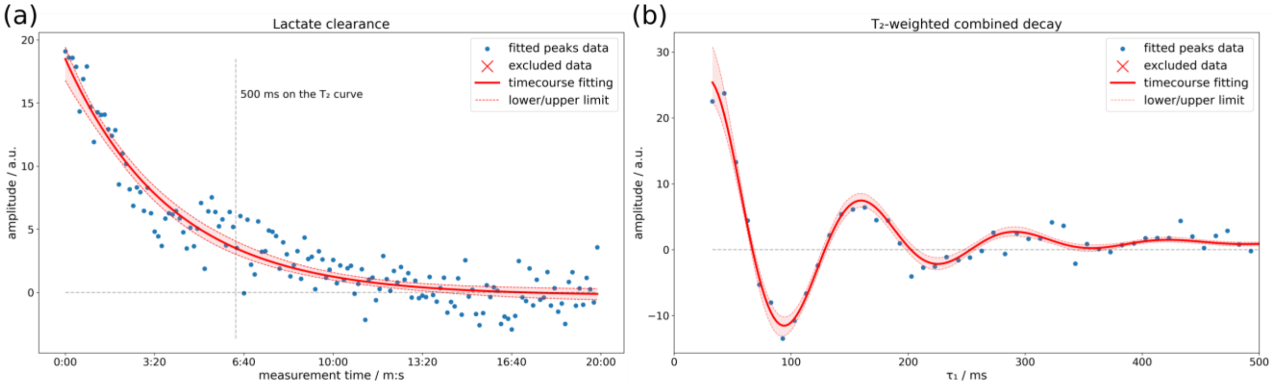

FIGURE S5. Data described in the paragraph above for subject № 2, re-invited (right leg).

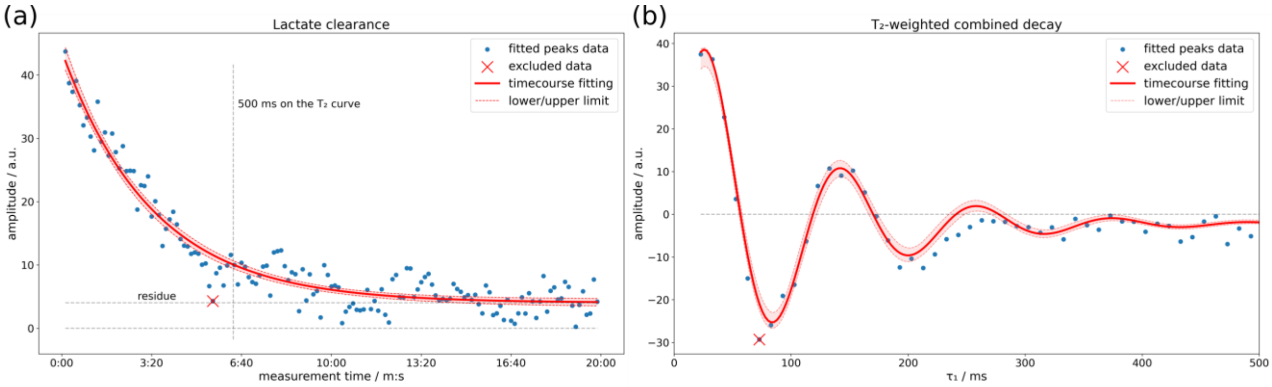

FIGURE S6. Data described in the paragraph above for subject № 3. The offset, of 10 % of the

total signal is presumably due to lipid residues at 1.3 ppm.

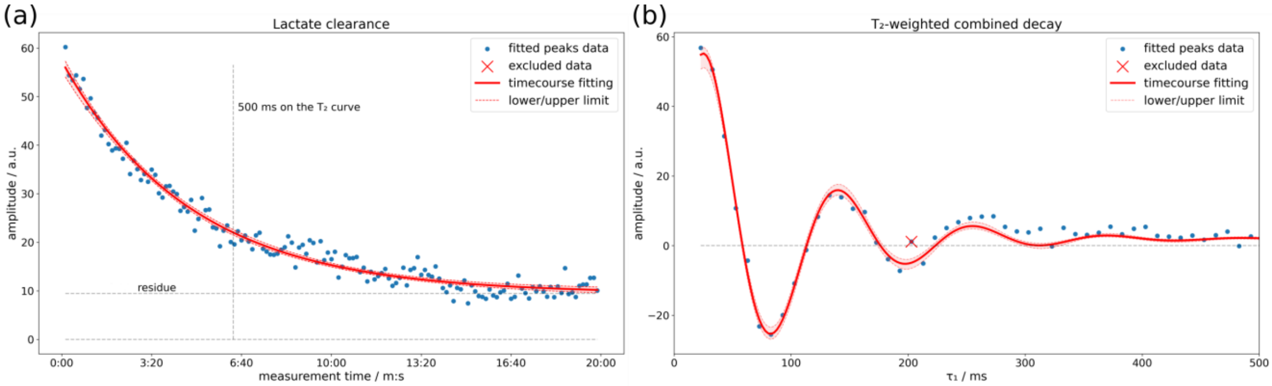

FIGURE S7. Data described in the paragraph above for subject № 4. The offset, of 20 % of the total signal is presumably due to lipid residues at 1.3 ppm.

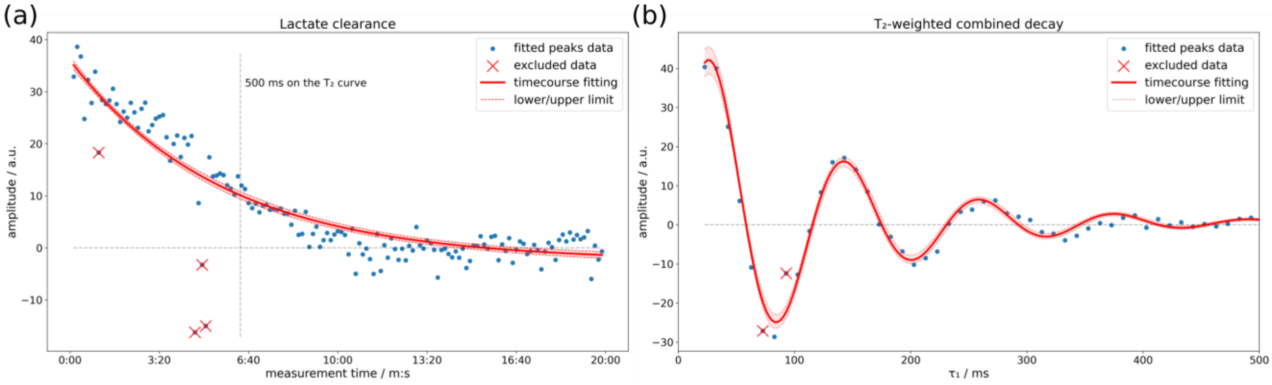

FIGURE S8. Data described in the paragraph above for subject № 6.

Figure S9 shows an exemplary spectrum acquired with the new sequence.

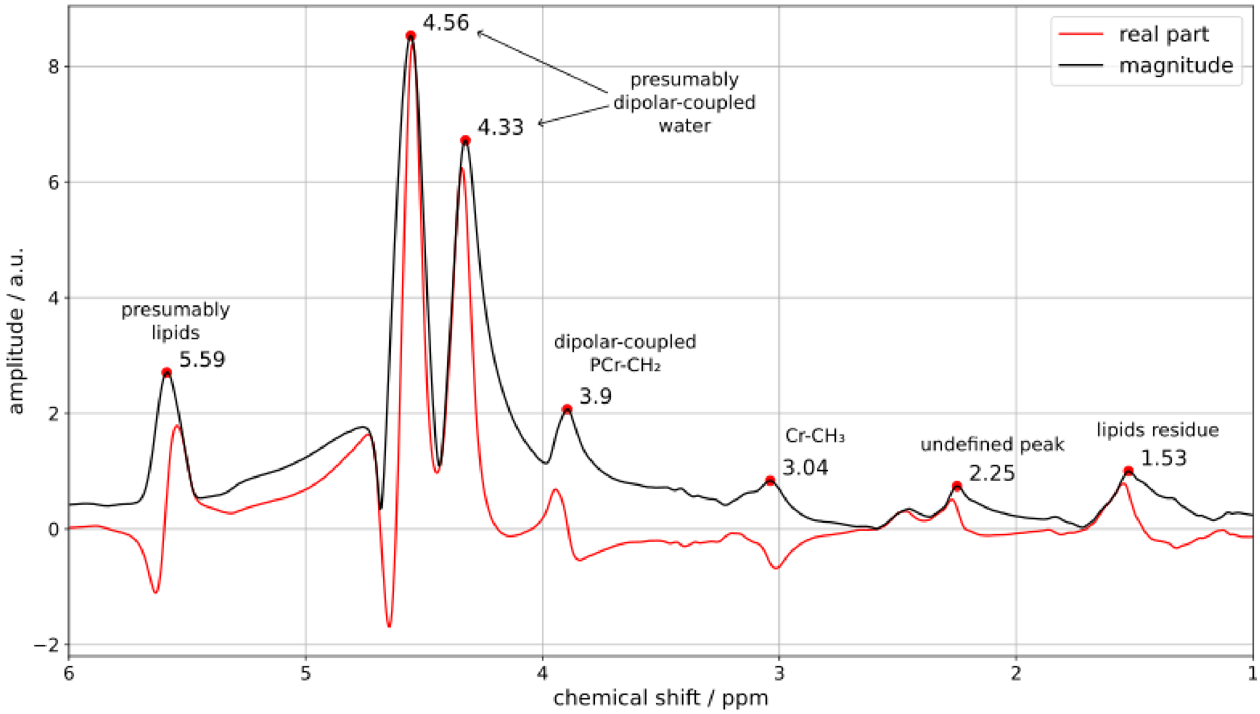

FIGURE S9. Typical DQF spectrum acquired in the subject № 4, averaged through the whole time-course (150 time points) in the lactate clearance dataset only. Spectra were not frequency or phase corrected before averaging, contributing in additional broadening of the peaks. Zero filling was (2×) and apodisation (10 Hz) were applied.
