# Supplementary material for: Postexercise Lactate Clearance, T 2 Relaxation, and J ‐Modulation in Human Skeletal Muscle Measured With Double‐Quantum Filtered 1H MRS at 7 T
Source: Magn Reson Med. 2026 Feb 10;95(6):3065–76. doi: 10.1002/mrm.70295 (PMC13049247; doi:10.1002/mrm.70295)
Supplement: Supplementary file 2 — Data S1. mrm70295‐sup‐0002‐supinfo. [file MRM-95-3065-s002.docx]

# Supplementary Material

# S1. Simulation of imperfect spoiling

**Methods**

To evaluate the impact of deviations between the predicted signal with perfect spoiling assumed and signal with imperfect spoiling, we performed additional simulations without perfect spoiling but RF and gradient spoiling. Based on the simulation described 3.1, an ensemble of 360 isochromats was simulated. Each isochromat represents a set of spins with identical initial conditions. After each RF excitation, the transverse magnetization vectors of all isochromats were summed to obtain the net macroscopic signals as would be measured by the scanner. During each TR, the isochromats underwent T1 and T2 decay. Instead of setting the transverse magnetization to zero at the end of each TR, gradient spoiling was simulated by rotating each isochromat with a distinct phase around the z-axis (ranging from 1° to 360° in 1° steps). To simulate RF spoiling, the phase of the RF pulse was incremented according to a linear scheme as described by Zur et al. (45).

First, for a FA of 5° and isochromats with T1 = 500 ms and T2 = 20 ms, spoiling angles of 117° and 150° were tested using both standard SPGR and the proposed I-SPGR sequence (TR = 5 ms for both SPGR and I-SPGR and T_FS_ = 100 ms, T_BS_ = 12 ms, and T_Sp_ = 10 ms for I-SPGR). The resulting signals were compared to the signals with perfect spoiling assumed. The simulations were repeated for a FA of 15°.

Secondly, the signal was simulated as a function of the spoiling angle for the same settings as stated above for both SPGR and I-SPGR acquisitions. For the I-SPGR case, the signal at the beginning of the shot (n = 1) was compared to the corresponding signal assuming perfect spoiling (also n = 1).

**Results**

At a low FA of 5° (Figure S1a), both SPGR signals with RF spoiling angles of 117° and 150° fluctuate slightly around the ideal SPGR signal obtained under perfect spoiling conditions. Similarly, the I-SPGR signals simulated with the same spoiling angles closely follow the ideal I-SPGR signal. Among them, the 150° spoiled signals show slightly better agreement with the perfectly spoiled signal than the 117° case, indicating more effective spoiling.
At a higher FA of 15° (Figure S1b), the influence of imperfect spoiling becomes more pronounced. The SPGR signal with 150° spoiling is lower than the theoretical SPGR signal, and the 117° spoiled signal is even lower. The I-SPGR signal shows more variation throughout the shot than SPGR, and the imperfectly spoiled versions fluctuate more noticeably from the ideal I-SPGR reference. Both imperfect spoiled signals over- and underestimate the perfectly spoiled signal during the shot.

When examining the signal as a function of the RF phase increment (spoiling angle) (Figure S1c), it can be observed that for a FA of 5°, the I-SPGR signal with RF and gradient spoiling overlaps with the one of perfect spoiling. In contrast, the SPGR sequence shows more irregular deviations from the expected signal, indicating higher sensitivity to the choice of spoiling angle.
At a FA of 15° (Figure S1d), the deviations between simulated and ideal signals increase for both sequences. The SPGR signal shows even more pronounced fluctuations across phase increments compared to the 5° case. This behavior is consistent with findings reported by Preibisch et al. (22) and Yarnykh et al. (46). Interestingly, the I-SPGR signal also exhibits larger deviations for specific phase increments but the deviations are smaller than the ones from the SPGR signal. The extent of deviation depends strongly on acquisition parameters (e.g., TR and FA), with different configurations leading to distinct signal patterns.


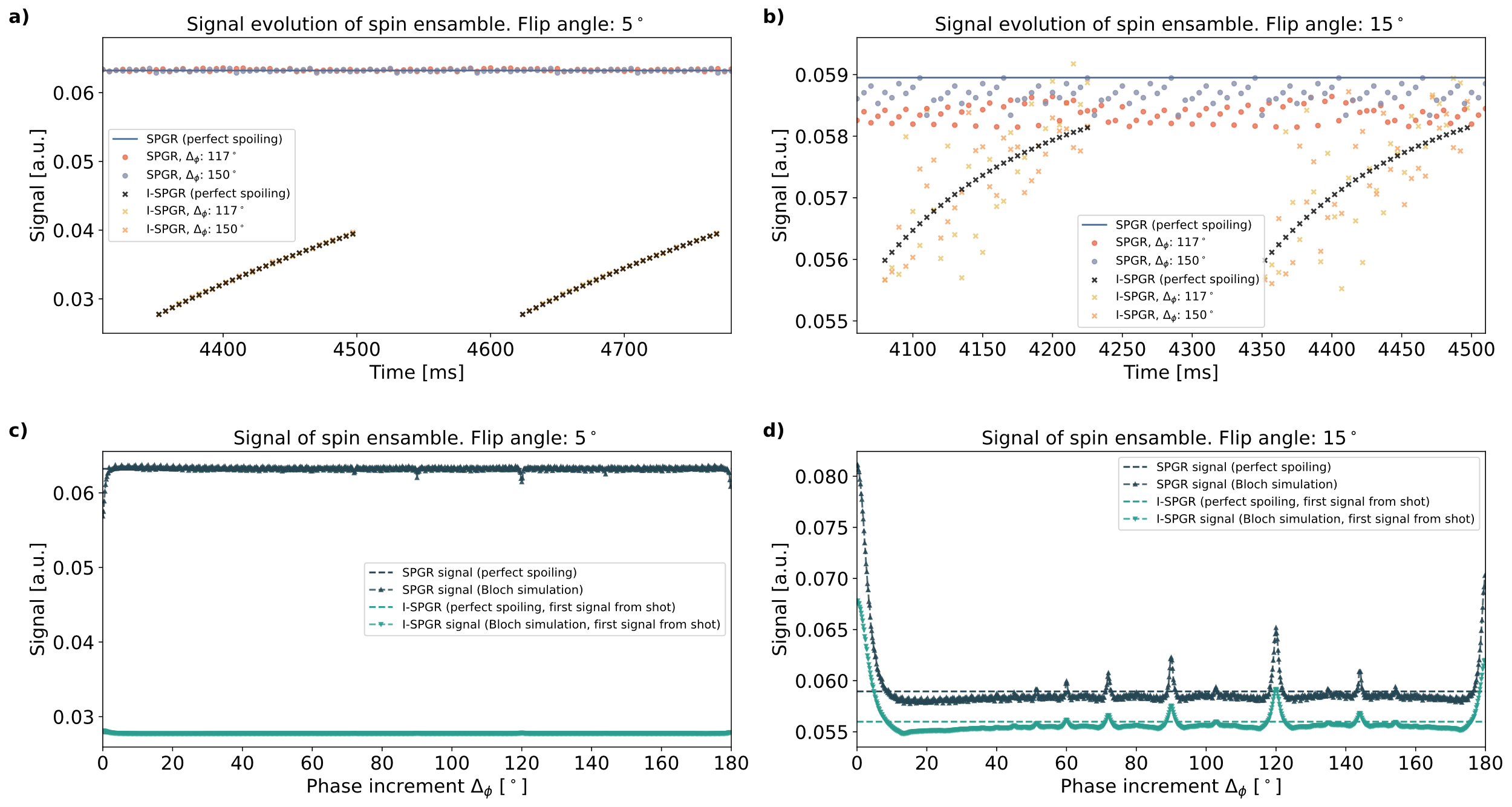


**Figure S1:** Comparison of SPGR and I-SPGR signals under varying RF spoiling conditions. a) Simulated signal evolution at a low flip angle (5°) shows almost no deviations from the signal with perfect spoiling. b) At a higher flip angle (15°), imperfect spoiling leads to more pronounced deviations, both for the SPGR and I-SPGR signals. c) Signal variation across RF phase increments at a flip angle of 5° shows I-SPGR to be robust, while SPGR exhibits irregular sensitivity to the spoiling angle. d) At a flip angle of 15°, both sequences show increased signal deviations, with I-SPGR displaying slightly less dependencies on the spoiling angle. All simulations are run with TR = 5 ms, T_FS_ = 100 ms, T_BS_ = 12 ms, and T_Sp_ = 10 ms and isochromats with T1: 500 ms and T2: 20 ms.

# S2. Sensitivity Analysis

**Methods**

We performed a simulation-based sensitivity analysis to assess the robustness and precision of the proposed I-SPGR sequence and signal model under noise. For different sets of T1 and T2 values the MRI signal was simulated using the actual acquisition settings of the I-SPGR sequence (see Table 1 for acquisition parameters). T1 values ranged from 100 to 3000 ms (10 steps), T2 values from 10 to 300 ms (10 steps), and M0 was fixed at 1000. Then additive Gaussian noise at a signal-to-noise ratio (SNR) of 30 was added to the simulated signals. SNR was defined as the maximum detected signal divided by the standard deviation of the noise. Each simulated signal was then fitted using the same model, and the fitted T1, T2, and M0 values were compared to the ground-truth values. This process was repeated 500 times per T1 and T2 combination to account for variability introduced by random noise, and the mean absolute error (MAE) was recorded.

For comparison, the same simulation and fitting procedure was applied to conventional SPGR signals generated with identical noise conditions and acquisition settings as given in Table 1. This allows for a comparison of the noise sensitivity between the proposed I-SPGR sequence and standard SPGR VFA approach.

We further examined the influence of acquisition timings by systematically varying TR and T_FS_. The minimum TR was set to 3.5 ms, and the total shot duration was constrained to be within the range of 300 ms to 600 ms to meet SAR limitations and maintain acceptable scan time. For each modified acquisition setting, the simulation and fitting procedure were repeated, and the MAE was calculated. We then identified the acquisition parameter set that minimized MAE.

**Results**

When Gaussian noise was added at an SNR of 30, the I-SPGR fitting yielded MAE of 21% for M0, 22% for T1, and 23% for T2 (Figure S2). Errors in the I-SPGR method were particularly high at low ground-truth T2 values (<40 ms), where T1 and M0 estimation errors exceeded 60%. This sensitivity to noise for low T2 likely reflects the relatively long T2 preparation times of the blood suppression pulses (12, 20, and 35 ms), during which signal decay severely reduces available signal for fitting. At larger T2 values, relative errors in T1 and M0 decreased to approximately 10–20%. T2 estimation errors peaked at ~50% when ground-truth T1 values fell below 1000 ms.

Under similar noise conditions the SPGR fitting achieved substantially lower errors, with MAE of 4% for M0 and 6% for T1 (Figure S3). These findings demonstrate that I-SPGR with the chosen acquisition settings is markedly more sensitive to noise than SPGR.

Acquisition timing had a notable impact on fitting accuracy. Optimizing for T1 fitting accuracy (TR = 19 ms, T_FS_ = 20 ms) reduced errors for both T1 and M0 to 12% (Figure S4). T2 errors (MAE 22%) remained similar to those in the baseline configuration. Alternatively, optimizing for T2 accuracy (TR = 8 ms, T_FS_ = 62 ms) yielded a T2 MAE of 21%, though errors still exceeded 60% at short T1 values. Under this configuration, T1 and M0 errors improved to MAEs of 17% and 18%, respectively, but did not reach the reductions observed with the T1-optimized parameters. Further improvements in T2 fitting accuracy may be possible by adjusting the blood suppression times, which strongly influence the T2 dependence of the signal.


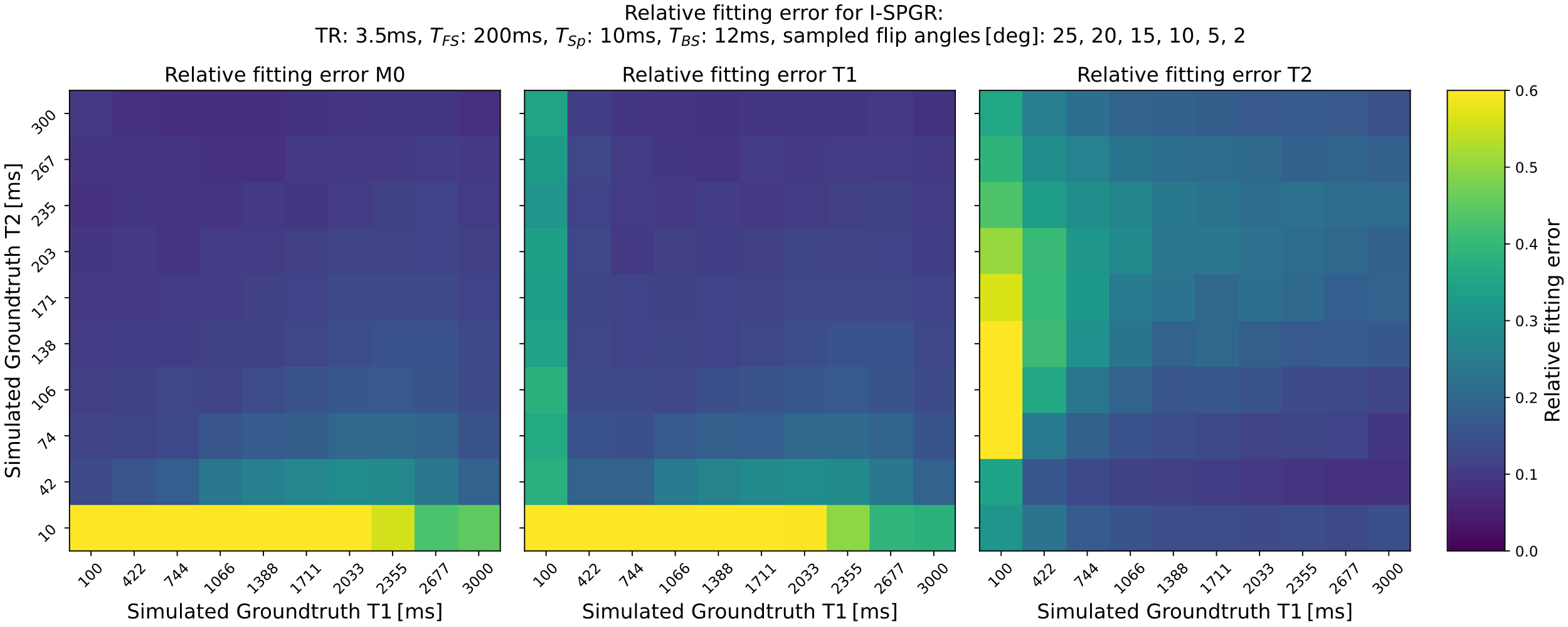


**Figure S2.** Fitting errors for the I-SPGR signal model under simulated noise conditions (SNR = 30). Unlike SPGR, I-SPGR exhibits substantial errors in T1, T2, and M0 estimation—particularly at low ground-truth T2 values (<40 ms), where T1 and M0 errors can exceed 60%. T2 estimation is also unreliable when T1 is below ~1000 ms, indicating greater sensitivity of I-SPGR to noise.


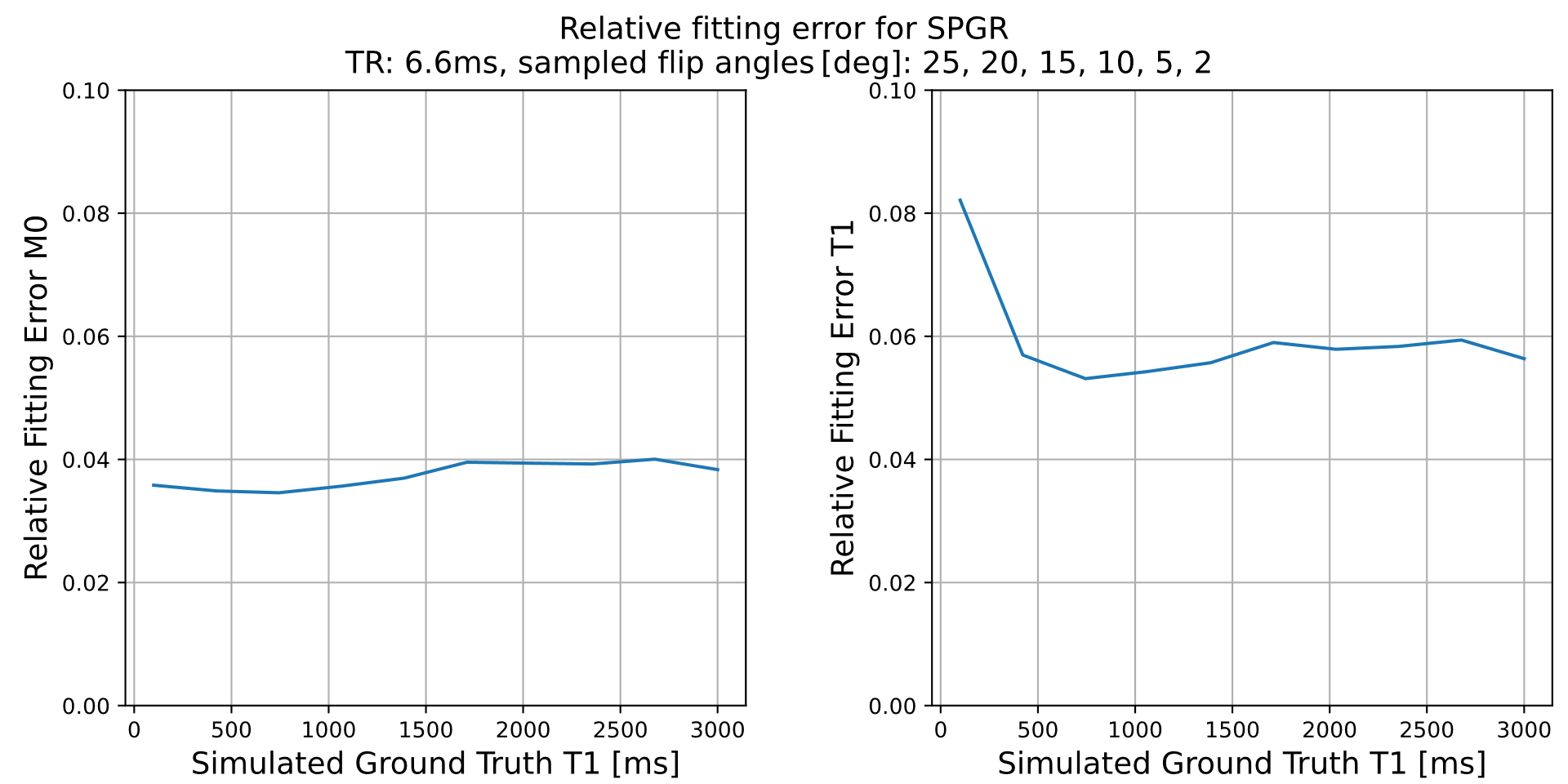


**Figure S3.** T1 and M0 fitting errors for the SPGR signal model under simulated noise conditions. With additive Gaussian noise (SNR = 30), SPGR maintains accurate estimation of T1 and M0 across a range of T1 and M0 values, showing only minor fitting errors. These results demonstrate SPGR's robustness to moderate noise.


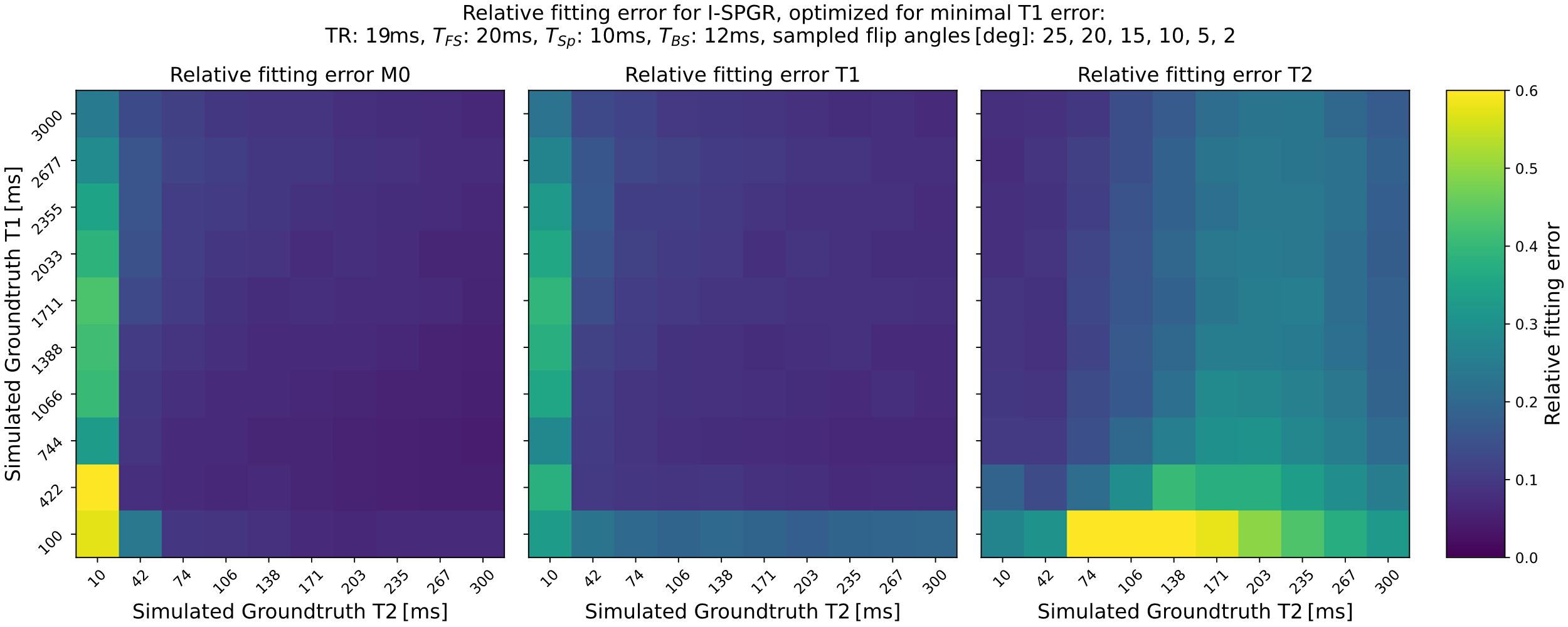

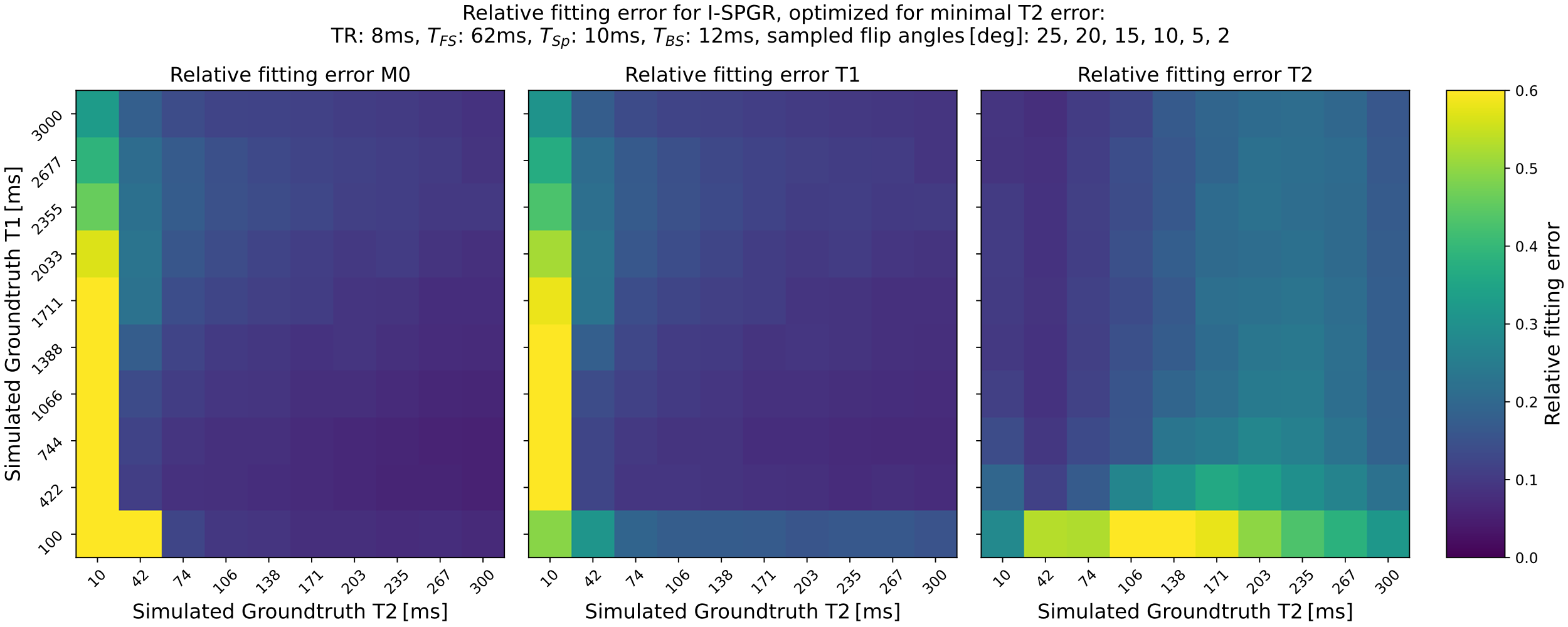


**Figure S4:** Relative fitting errors for T1, T2, and M0 using optimized acquisition parameters for the I-SPGR sequence. Top row: The T1-optimized configuration (TR = 19 ms, T_FS_ = 20 ms) substantially reduced fitting errors in T1 and M0, with moderate improvement in T2 estimation—though errors at low T2 values still reached ~40%. Bottom row: In contrast, optimizing for minimal T2 error (TR = 8 ms, T_FS_ = 62 ms) yielded the lowest T2 errors, particularly at higher T1 values, while T1 and M0 errors improved modestly compared to the original acquisition but remained higher than in the T1-optimized case.

# S3. Repeatability and Reproducibility Analysis

**Results:**

**Phantom data:**

Same-day repeatability (Figure S5, left column) showed highly stable measurements across all sequences, with mean differences of −4.4 ms for SPGR T1, −1.5 ms for I-SPGR T1, and −1.4 ms for I-SPGR T2. The 95% limits of agreement were narrow (typically within ±10–15 ms), although the confidence interval for T1 I-SPGR was slightly larger than that of SPGR. In contrast, for T2 the I-SPGR implementation showed a smaller confidence interval. Repeatability wCV values were low (≤0.01). Between-day reproducibility (Figure S2, right column) also remained high, with wCV values ≤0.02 across all phantom compartments. These results confirm that both T1 and T2 estimates obtained with SPGR and I-SPGR are consistent across repeated acquisitions and stable across days.


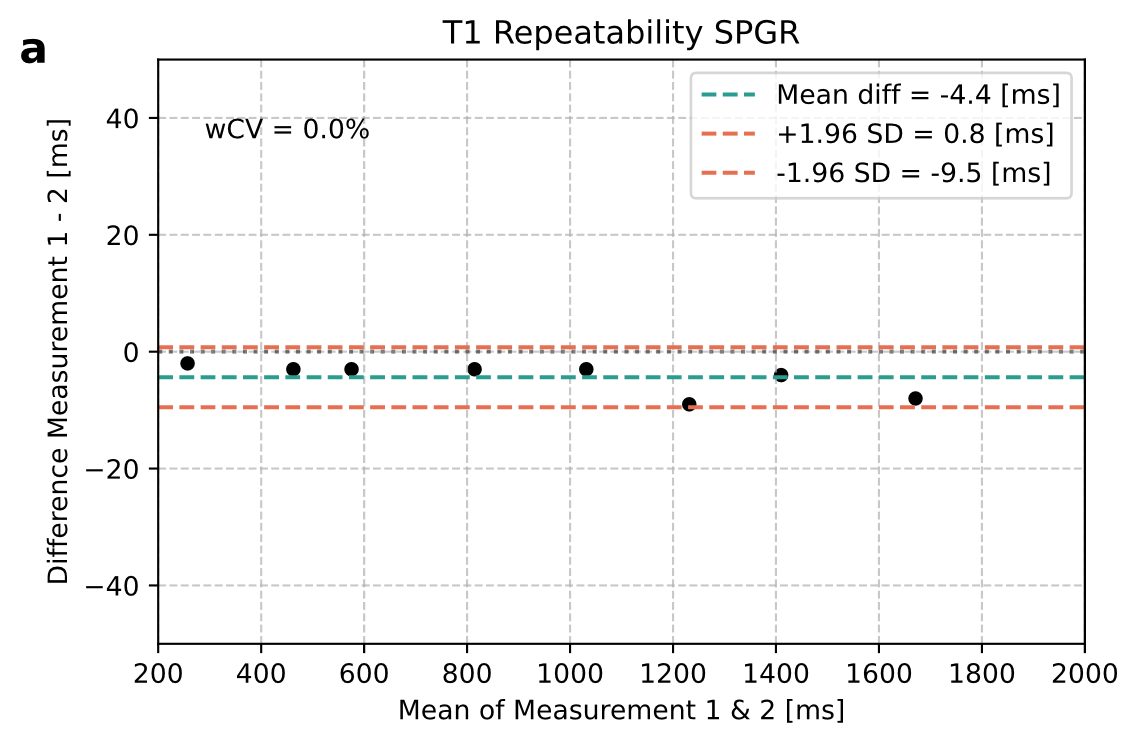

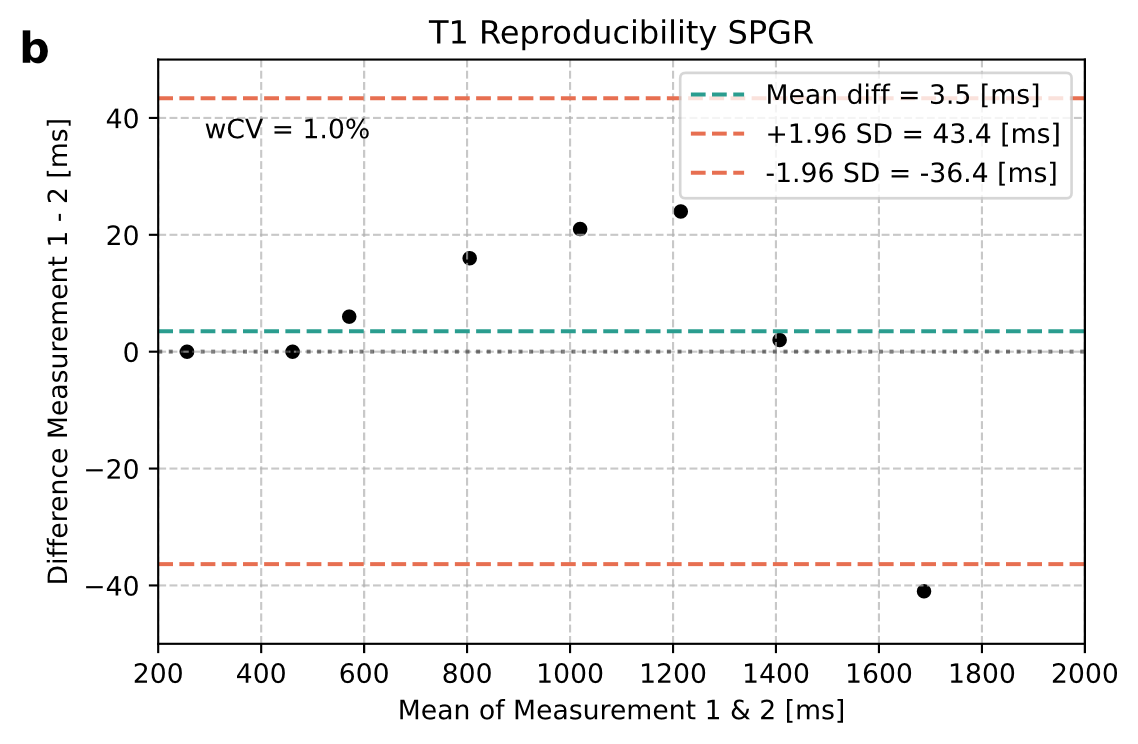

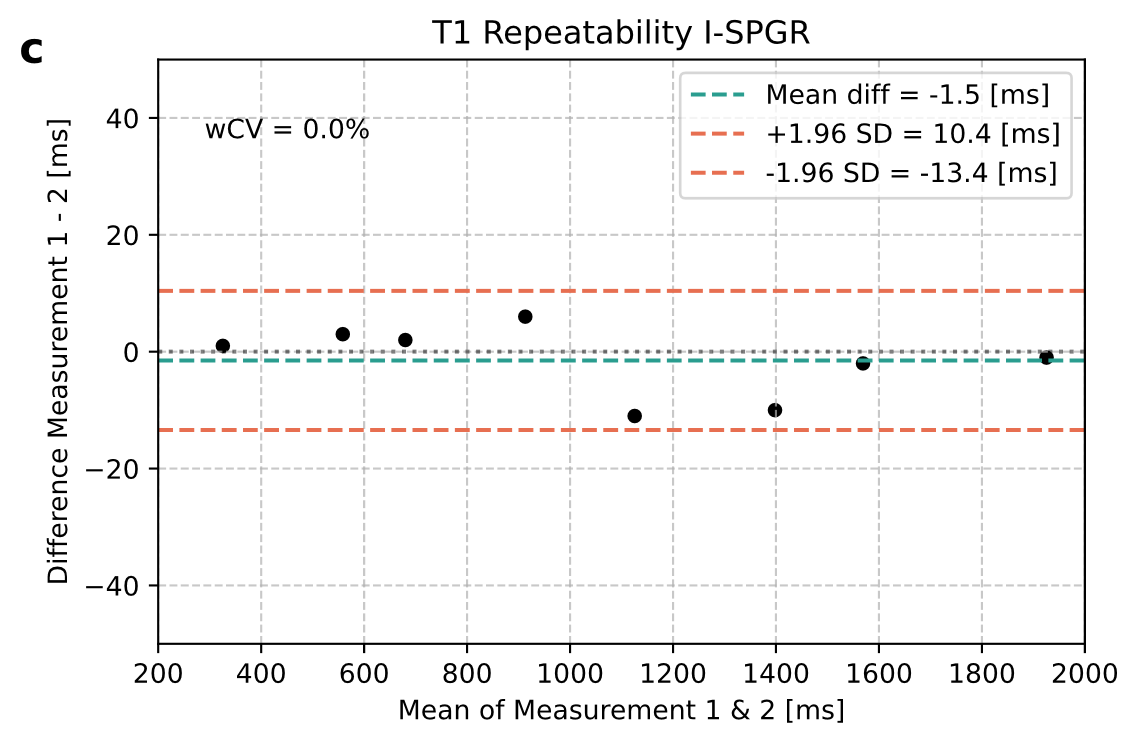

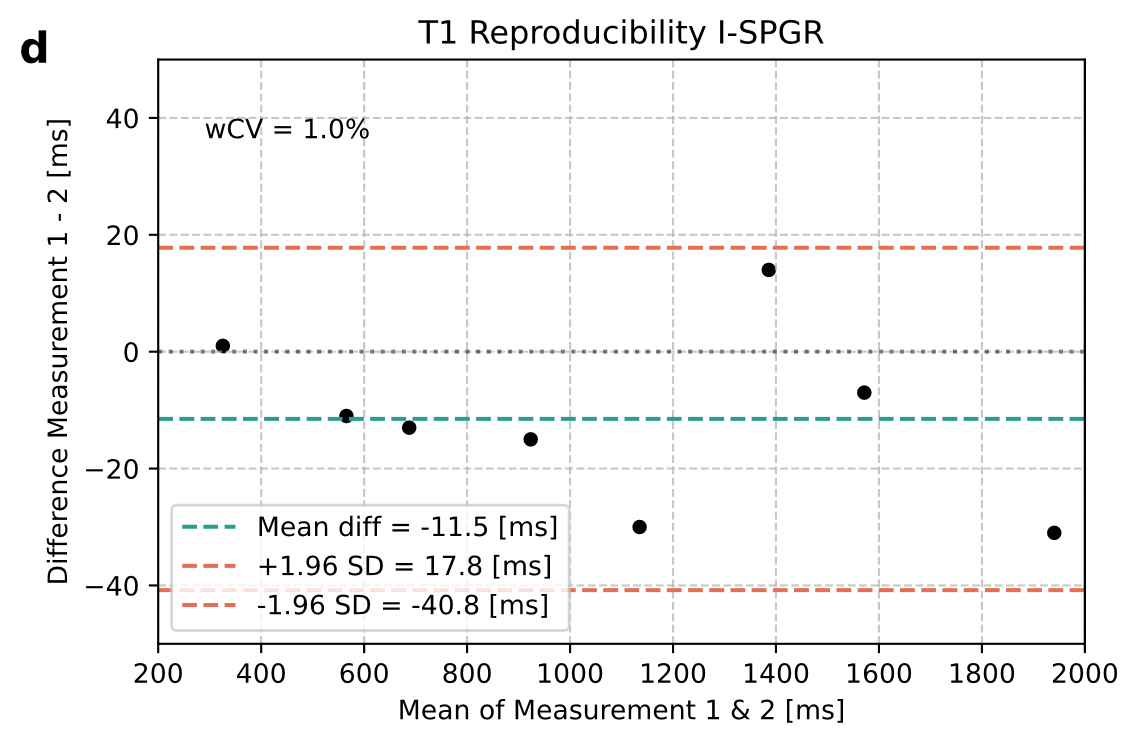

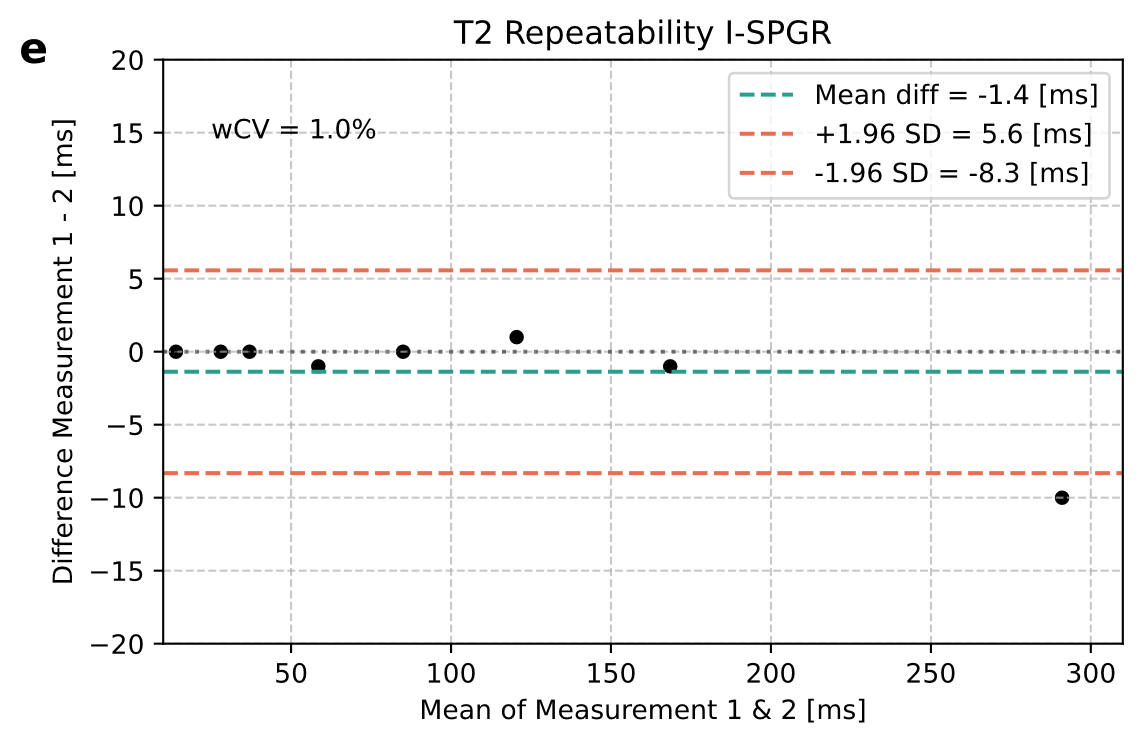

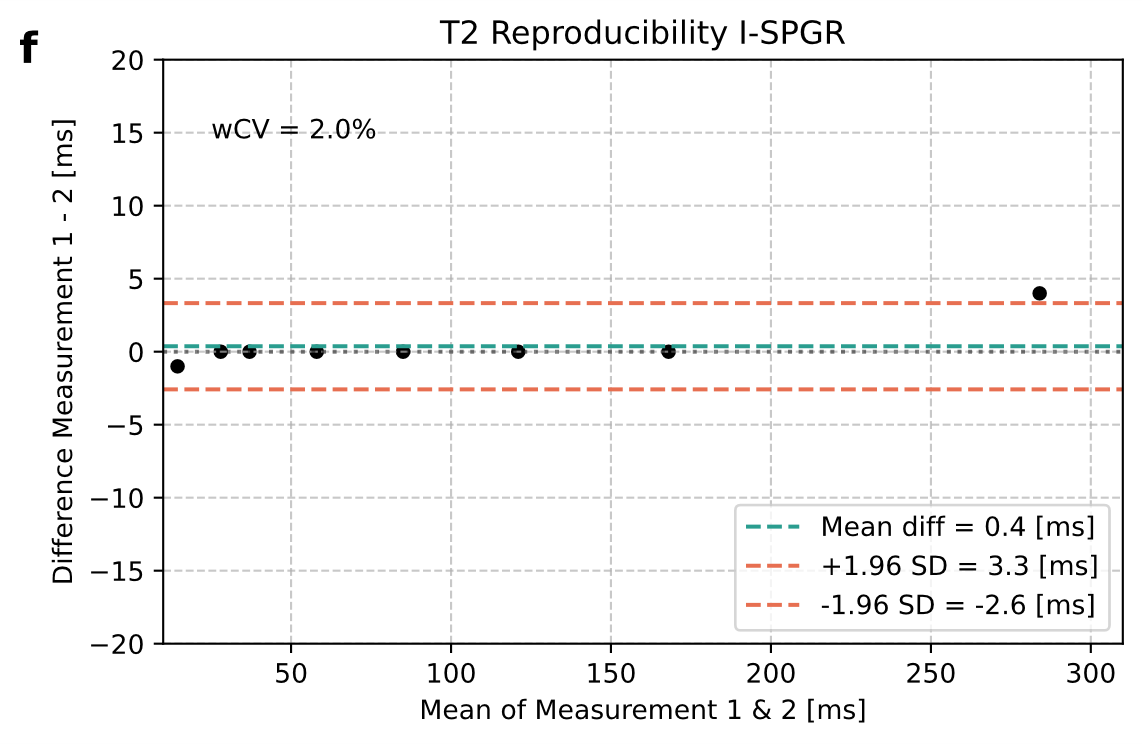


**Figure S5**: Same day repeatability (a, c, e) and reproducibility (b, d, f) of T1 and T2 measurements in the phantom for SPGR T1 (a–b), I-SPGR T1 (c–d), and I-SPGR T2 (e–f). Bland–Altman plots show the difference between repeated measurements (Measurement 1 – Measurement 2) as a function of their mean, along with the mean difference (dashed green line) and the 95% limits of agreement (±1.96 SD; dashed red lines). Within-subject coefficient of variation (wCV) values are reported for each sequence.

**In vivo data:**

Same-day in vivo repeatability was assessed in seven healthy volunteers using the I-SPGR sequence. Due to the small thickness of the aortic wall, quantitative analysis was performed in the psoas muscle, which provides a larger and more homogeneous region of interest (Figure S6). Bland–Altman analysis demonstrated stable measurements over time (Figure S7). The corresponding within-subject coefficients of variation (wCV) were 0.11 for T1 and 0.05 for T2, further supporting higher repeatability for T1 and T2 measurements in vivo. Mean T1 values were ranging from 1387 ms to 1756 ms and mean T2 values ranging from 32 ms to 51 ms.


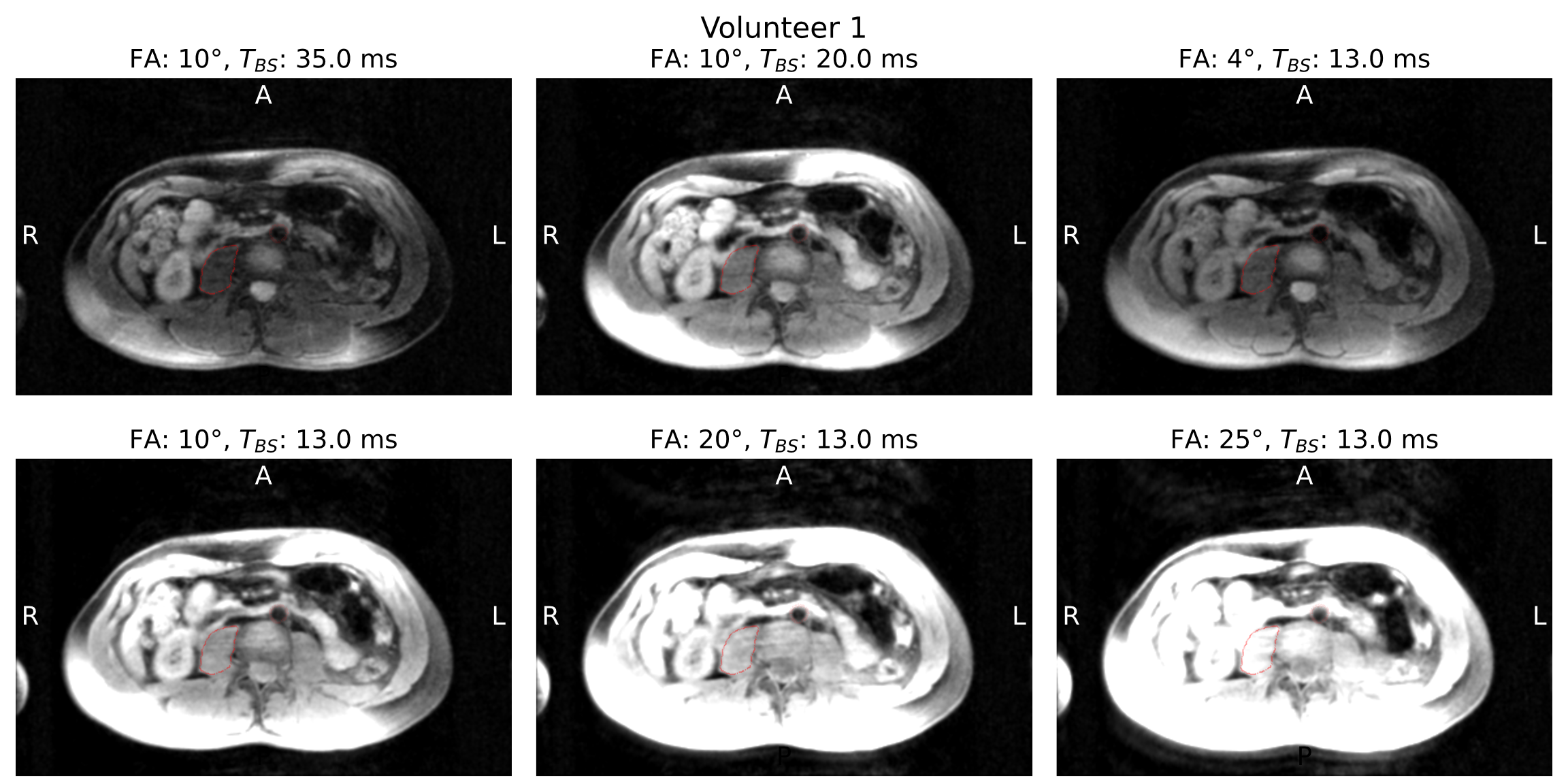


**Figure S6:** Representative I-SPGR images from one volunteer with psoas muscle and aortic wall indicated (red). Note that to show signal changes over FAs, window-level was kept the same, resulting in some images being saturated and others being low in intensity.


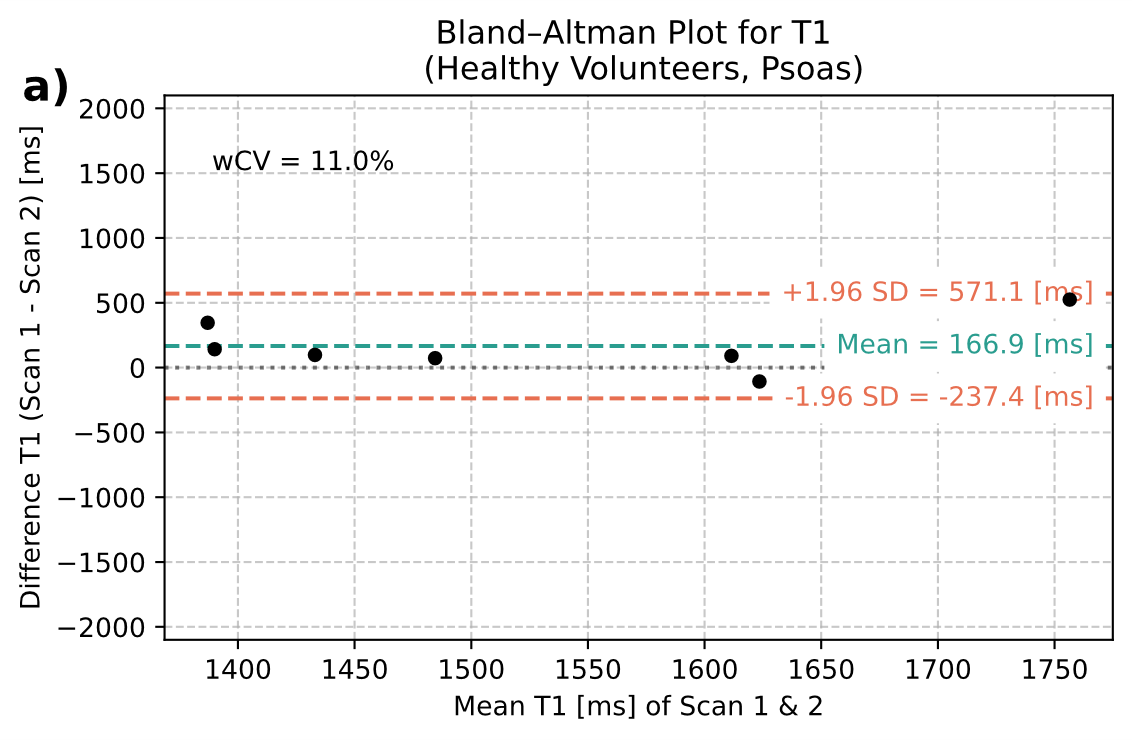

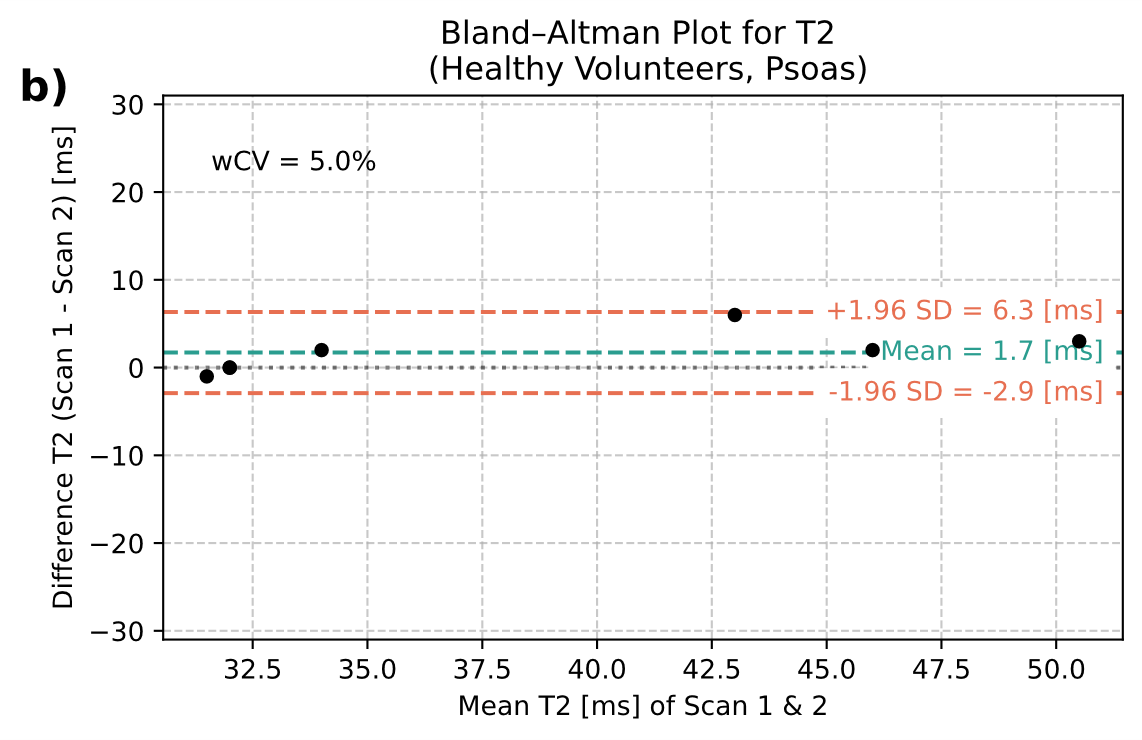


**Figure S7**: Bland–Altman analysis of T1 (a) and T2 (b) measurements obtained from repeated same-day healthy volunteer acquisitions using the I-SPGR sequence. Plots show the difference between measurements (Median T1 or T2 scan 1 – Median T1 or T2 scan 2) versus their mean (1/2 x Median T1 or T2 scan 1 + ½ x Median T1 or T2 scan 2), along with the mean difference (dashed green line) and 95% limits of agreement (±1.96 SD; dashed red lines). Within-subject coefficient of variation (wCV) values are reported.
